# Beta cell microRNAs function as molecular hubs of type 1 diabetes pathogenesis and as biomarkers of diabetes risk

**DOI:** 10.1007/s00125-026-06720-7

**Published:** 2026-04-21

**Authors:** Farooq Syed, Preethi Krishnan, Garrick Chang, Jyoti Rana, Sarah R. Langlais, Sumon Hati, Kentaro Yamada, Anh K. Lam, Sayali Talware, Jacqueline Del Carmen Aquino, Eli Hagedorn, Xiaowen Liu, Rajesh Sardar, Jing Liu, Raghavendra G. Mirmira, Carmella Evans-Molina

**Affiliations:** 1 Department of Pediatrics, Indiana University School of Medicine, Indianapolis, IN USA; 2 Center for Diabetes and Metabolic Diseases, Indiana University School of Medicine, Indianapolis, IN USA; 3 Herman B Wells Center for Pediatric Research, Indiana University School of Medicine, Indianapolis, IN USA; 4 Department of Diabetes-Immunology, Arthur Riggs Diabetes & Metabolism Research Institute, City of Hope, Duarte, CA USA; 5 Center for RNA Biology and Therapeutics, Beckman Research Institute, City of Hope, Duarte, CA USA; 6 Department of Medicine, Indiana University School of Medicine, Indianapolis, IN USA; 7 Department of Physics, Indiana University Indianapolis, Indianapolis, IN USA; 8 Department of Chemistry and Chemical Biology, Indiana University Indianapolis, Indianapolis, IN USA; 9 School for Informatics and Computer, Indiana University Indianapolis, Indianapolis, IN USA; 10 Deming Department of Medicine, Tulane University School of Medicine, New Orleans, LA USA; 11 Department of Physics and Astronomy, Purdue University, West Lafayette, IN USA; 12 Kovler Diabetes Center, University of Chicago, Chicago, IL USA; 13 Roudebush VA Medical Center, Indianapolis, IN USA

**Keywords:** Beta cell, Biomarkers, Extracellular vesicles, Localised surface plasmon resonance, microRNA, Type 1 diabetes

## Abstract

**Aims/hypothesis:**

Clinically actionable biomarkers that accurately reflect the health status of the beta cell are needed to improve risk stratification and optimise the timing of interventions in type 1 diabetes. We hypothesised that inflammatory stress elicits a reproducible microRNA (miRNA) program in human islets and islet-derived extracellular vesicles (EVs) that can be detected in plasma EVs to stratify diabetes risk, while also providing insight into molecular pathways linked to beta cell dysfunction.

**Methods:**

Human islets were exposed to IL-1β+IFN-γ, and small RNA-seq was performed on islets and islet-derived EVs. Differentially expressed miRNAs were validated in islets, using RT-PCR, in plasma-derived EVs from individuals with autoantibody positivity (AAb^+^) or recent-onset type 1 diabetes and matched control individuals using ultrasensitive, label-free localised surface plasmon resonance (LSPR) biosensors, and in pancreatic sections from organ donors using in situ hybridisation and spatial feature analysis. Finally, beta cell-targeted in vivo inhibition of miR-155 was tested in the NOD mouse model.

**Results:**

Inflammatory cytokine exposure altered a restricted subset of miRNAs, identifying 20 differentially expressed miRNAs in islets and 14 in islet-derived EVs. Only two miRNAs, miR-155-5p and miR-146a-5p, were concordantly upregulated in both compartments. Machine learning prioritised an EV miRNA panel for translational validation, and custom LSPR biosensors enabled quantification of these miRNAs in plasma EVs. This plasma EV miRNA signature, consisting of miR-155-5p, miR-146a-5p, miR-30c-1-3p, miR-802 and miR-124-3p, differentiated individuals with AAb^+^ and those with recent-onset type 1 diabetes from control individuals with good sensitivity and specificity. In pancreatic tissue, miR-155 abundance and beta cell spatial/subcellular distribution were altered in donors with AAb^+^ and type 1 diabetes compared with non-diabetic control individuals. Functionally, beta cell-targeted inhibition of miR-155 improved glucose tolerance and reduced insulitis in prediabetic NOD mice.

**Conclusions/interpretation:**

Using an organ-based model system of inflammatory stress, we validated a signature of EV-associated miRNAs capable of stratifying type 1 diabetes risk. Furthermore, we provided new mechanistic and imaging insights into miRNA expression patterns in pancreatic sections from human organ donors with type 1 diabetes or AAb^+^, and we used a preclinical model of type 1 diabetes to demonstrate the potential therapeutic efficacy of targeting these miRNAs.

**Data availability:**

The data from small RNA sequencig of human islets and islet-derived EVs have been deposited in the GEO database (accession no. GSE160391).

**Graphical Abstract:**

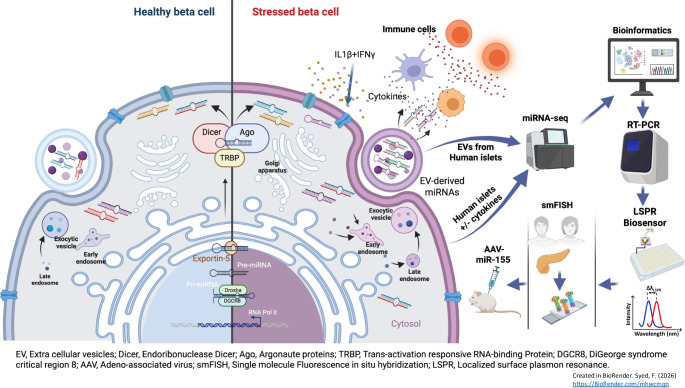

**Supplementary Information:**

The online version contains peer-reviewed but unedited supplementary material available at 10.1007/s00125-026-06720-7.


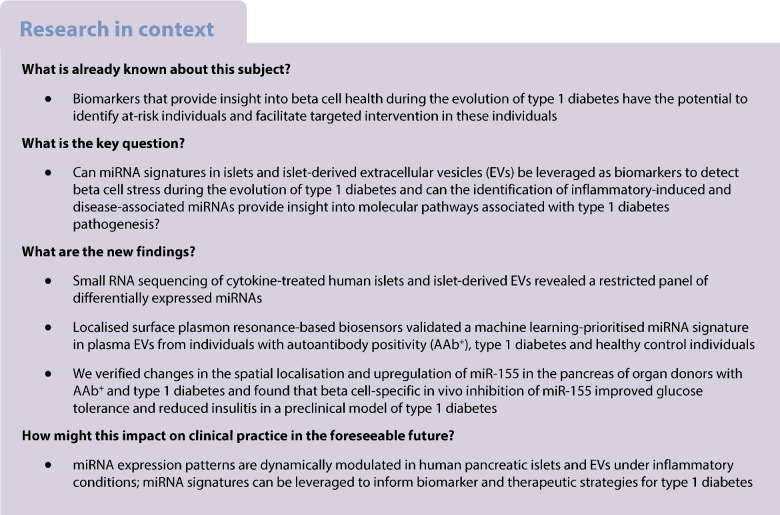


## Introduction

Type 1 diabetes results from immune-mediated destruction of pancreatic beta cells [1]. Data from natural history studies enrolling newborns with high genetic risk indicate that nearly 70% of individuals with two or more type 1 diabetes-associated autoantibodies (AAbs) will develop clinical disease within 10 years of follow-up [2]. These epidemiological data formed the basis for a new staging paradigm introduced over a decade ago, where stage 1 type 1 diabetes is defined as the presence of two or more islet AAbs and normal glucose tolerance, stage 2 disease is defined as the presence of multiple islet AAbs and dysglycaemia and stage 3 type 1 diabetes is marked by the development of hyperglycaemia that exceeds the ADA thresholds for clinical diagnosis [3–7]. Immunomodulatory interventions initiated at the onset of stage 3 type 1 diabetes have shown limited efficacy in inducing a durable disease remission [4–8]. However, teplizumab, an Fc-receptor-non-binding anti-CD3 monoclonal antibody, delayed the onset of stage 3 type 1 diabetes by approximately 32 months in high-risk individuals with stage 2 type 1 diabetes [9, 10]. Teplizumab was approved by the US Food and Drug Administration (FDA) in November 2022 as the first disease-modifying therapy for type 1 diabetes.

While results from the teplizumab trial provided the first evidence that immune interventions are able to substantially alter the course of type 1 diabetes, variable responses to disease-modifying therapies have been observed in all clinical trials performed to date [11]. In addition, not all individuals with AAbs will progress to clinical disease, and not all AAbs are associated with the same risk and rate of disease progression [12]. Thus, the timeframe of type 1 diabetes development in at-risk individuals is highly variable, leading to challenges in the selection and timing of therapeutic interventions. Biomarkers capable of stratifying heterogeneous at-risk populations, especially markers that provide insight into the health status of the beta cell, have the potential to identify individuals most likely to benefit from early immunomodulatory intervention while also pointing to potentially actionable pathways associated with disease pathogenesis.

miRNAs are a class of endogenously produced and highly conserved small non-coding RNAs that are ~22 nucleotides in length. miRNAs are most commonly associated with inhibition of gene expression through complementary binding to the 3′ untranslated region of mRNAs to either repress mRNA translation or cause mRNA degradation. A broad range of biological processes within the beta cell are regulated by miRNAs, including insulin secretion, endoplasmic reticulum (ER) stress and apoptosis [13–16]. In addition, recent evidence suggests that miRNAs are enriched in extracellular vesicles (EVs), a class of membrane-bound vesicles that serve as important mediators of intracellular communication and thus represent potential biomarker targets [17–19]. miRNAs and EV-associated miRNAs can be detected in a variety of body fluids, including serum, plasma, urine, saliva and breast milk [17–22]; however, translating the measurement of miRNAs into the clinical trial setting requires extensive validation as well as the development of robust and high-throughput detection assays.

In this study, we hypothesised that miRNA signatures of islets and islet-derived EVs could be leveraged as biomarkers to detect ongoing beta cell stress during the evolution of type 1 diabetes, while also offering insight into beta cell pathways linked with disease development.

## Methods

### Human islet samples and human plasma samples

Islets isolated from a total of 32 de-identified organ donors were obtained from the Integrated Islet Distribution Program (IIDP; https://idp.coh.org). Islet donor and isolation characteristics, including donor age, sex, BMI, cause of death, measurements of islet purity and viability, ischaemia duration and culture time are provided in the human islet checklist (electronic supplementary material [ESM] Table 1). Upon receipt, islets were placed in EV-depleted standard Prodo islet media (Prodo Labs, CA, USA) and allowed to recover overnight. To investigate the effect of inflammation on islet miRNAs, islets were cultured with or without 50 U/ml IL-1β and 1000 U/ml IFN-γ for 24 h. Untreated and cytokine-treated human islets and islet cell supernatant fractions were harvested for downstream applications. For EV isolation, supernatant fractions were collected and centrifuged for 30 min at 3000 *g* to remove cellular debris, and EVs were then isolated using ExoQuick-TC (SBI Bioscience, USA), as per the manufacturer’s instructions.

Citrated plasma was obtained by venipuncture or withdrawal from an existing i.v. line from individuals with recent-onset type 1 diabetes and non-diabetic control individuals with similar mean age-, sex- and BMI. Samples from individuals with type 1 diabetes were obtained within 48 h of hospital admission for a new diagnosis of diabetes. Plasma samples from participants with diabetes-associated autoantibody positivity (AAb^+^) from the TrialNet Pathway to Prevention Study were collected under an approved ancillary study. All samples were processed and aliquoted within 1 h of collection and then stored at −80°C in a Biorepository maintained at the Indiana University School of Medicine. Written informed consent or parental consent or child assent was obtained from all participants prior to specimen collection. Collections were approved by the Indiana University School of Medicine Institutional Review Board, and investigations were carried out in accordance with the principles of the Declaration of Helsinki. For all participants, data on biological sex was collected by self-report; data for gender was not collected. Given the modest sample size in each group, the impact of biological sex on measured outcomes was not individually assessed.

### Scanning electron microscopy and transmission electron microscopy imaging analysis of islets and EVs

Scanning electron microscopy (SEM) and transmission electron microscopy (TEM) were performed at the Northwestern University Atomic and Nanoscale Characterisation Experimental Center (NUANC) in Evanston, IL, USA. Additional detail is provided in the ESM Methods.

### Nanoparticle tracking analysis

To determine the size and concentration of EVs, nanoparticle tracking analysis (NTA) (Particle Metrix, 82266 Inning am Ammersee, Germany) was used. Briefly, EVs isolated from human islet culture supernatant fractions were diluted 1:3000 in double-distilled H_2_O, and 1 ml of the diluted sample was subjected to NTA. Results were analysed using ZetaView Analysis software (Particle Metrix).

### Microarray chip-based tetraspanin staining

To determine the composition of EV cargo, we used ExoView microarray chips (NanoView Bioscience, ExoView, EV-TETRA-C-T) printed with antibodies for the tetraspanin markers CD63, CD81 and CD9 chips (ExoView Human Tetraspanin kit, 251-1000, NanoView Bioscience). Staining was performed according to the directions provided by the manufacturer. Additional detail is provided in the ESM Methods.

### Western blot analysis

EVs were washed twice with PBS and lysed with a buffer containing 50 mmol/l Tris, 150 mmol/l NaCl, 0.05% (wt/vol.) deoxycholate, 0.1% (wt/vol.) octylphenoxyethooxyethanol (IGEPAL), 0.1% (wt/vol.) Sodium dodecyl sulfate (SDS), 0.2% (wt/vol.) sarcosyl, 5% (wt/vol.) glycerol, 1 mmol/l dithiothreitol (DTT), 1 mmol/l ethylenediaminetetraacetic acid (EDTA), 10 mmol/l NaF and a cocktail of protease inhibitors (Roche Diagnostics, Germany) and phosphatase inhibitors (Roche Diagnostics, Germany). Protein concentrations were determined using the Lowry method (Bio-Rad), as per the manufacturer’s instructions. A total of 10 μl of protein was loaded onto 4–20% precast Tris-glycine gels (Bio-Rad) and electroporated at 100 V. Protein transfer was performed using a PVDF membrane (Millipore). Blots were blocked with LI-COR PBS-blocking buffer and probed with the following primary antibodies at 4°C: anti-CD63 (1:1000 dilution; SC13118, Santa Cruz, mouse monoclonal, RRID AB_627213); and anti-CD9 (1:1000 dilution; SC5275, Santa Cruz, mouse monoclonal, RRID AB_627877). Anti-rabbit or anti-mouse (1:10000) secondary antibodies were used for signal detection and band intensity was quantified using ImageStudio (LI-COR).

### RNA isolation from human islets and human islet-derived EVs

Total RNA was isolated from human islets using the miRNeasy Mini Kit (Qiagen, Germany) according to the manufacturer’s instructions. RNA quality and concentration were measured using 260/280 ratios (IMPLANT spectrophotometer, Germany), followed by assessment using the Agilent Bioanalyzer 2100 and small RNA assay chips (Agilent Technologies, USA). Exosomal RNA was isolated using the SeraMir RNA isolation kit (System Biosciences, RA806A-1) according to the manufacturer’s instructions. Briefly, isolated EVs were lysed with 350 µl SeraMir lysis buffer by vortexing for 15 s and allowing the lysate to stand at room temperature for 5 min. Next, 200 µl of 100% ethanol was added to the lysate and mixed by vortexing for 10 s. The lysate was transferred to a spin column and centrifuged at 15,871 *g* for 1 min. The flow-through was discarded and the column was washed twice with 400 µl of wash buffer (SeraMir, System Bioscience), followed by centrifugation at 15,871 *g* for 1 min. Finally, the columns were dried by centrifugation at 15,871 *g* for 2 min, and RNA was eluted by adding 30 µl of elution buffer (SeraMir, System Bioscience).

### Small RNA sequencing

Total RNA and miRNA were first evaluated using the Agilent Bioanalyzer for quantity, quality and per cent miRNA content in total RNA. The starting amount of RNA was 10–20 ng for each miRNA library. For miRNA library preparation, 0.5% of miRNA in total RNA was the cut-off to decide whether the sample should be enriched for miRNA. When needed, enrichment was performed following the small RNA library preparation procedure and the Ion Total RNA-Seq Kit v2 User Guide, Pub. no. 4,476,286 Rev. E (Life Technologies, USA). Each resulting barcoded library was quantified and assessed for quality using the Agilent Bioanalyzer. Multiple libraries were pooled in equal molarity. Eight microlitres of 100 pmol/l pooled libraries were applied to the Ion Sphere Particles (ISP) template preparation, and amplification was achieved using the Ion OneTouch 2, followed by ISP loading onto PI chips and sequencing using the Ion Proton semiconductor. Each PI chip is capable of generating approximately 50 million usable reads of 21–22 bp miRNA fragments. Sequence mapping was performed using Torrent Suite Software v4.6 (TSS 4.6). For details on miRNA sequence data analysis, see ESM Methods.

### Selection of internal reference genes for quantitative reverse-transcription PCR for miRNAs

Potential reference miRNAs capable of serving as normalisation strategies for quantitative reverse-transcription PCR (qRT-PCR) were identified using sequencing data from human islets and islet-derived EVs. See ESM Methods for additional details.

### cDNA synthesis and qRT-PCR for mRNA and miRNAs

To determine the expression of genes involved in EV biogenesis, 0.5–1 µg of total RNA was isolated from human islets and reverse transcribed using an M-MLV RT kit (Invitrogen, MA, USA). See ESM Methods for additional details.

### Feature selection and machine learning analysis to identify predictive miRNA signatures

For feature selection, we used Learning Vector Quantification (LVQ) implemented in the ‘caret’ R package to compute the importance values of features. See ESM Methods for additional details.

### Measurement of human plasma-derived EV miRNAs using localised surface plasmon resonance-based biosensors

Details of EV isolation from human plasma are provided in the ESM Methods (Preparation of human plasma-derived EVs and RNA isolation). Total RNA was isolated from human EVs using the SeraMir Exosome RNA purification kit (RA808A-1, System Biosciences, Palo Alto, CA, USA), according to the manufacturer’s instructions. RNA quality of each sample was measured using NanoDrop (Thermo Scientific, USA) and the Agilent Bioanalyzer Pico Assay using the Bioanalyzer 2100 Expert instrument (Agilent Technologies, Santa Clara, CA, USA). A custom LSPR-based biosensor was developed as described in the ESM Methods (see LSPR-based biosensor construction). Synthetic mature miRNAs (ESM Table 2) were used as a positive control to gate thresholds, and the data were presented as the concentration of miRNA (fmol/l to nmol/l).

### EndoC-βH1 EV analysis

Isolated EVs were processed for total RNA isolation using the miRNeasy RNA purification kit (217004, Qiagen), according to the manufacturer’s instructions provided with the kit. See ESM Methods for additional details.

### Functional prediction of validated miRNAs

Functional enrichment analysis of the five selected miRNAs was carried out using DIANA miRPath v.3 [23]. See ESM Methods for additional details.

### BaseScope and RNAscope plus smRNA-RNA assays

To define tissue expression patterns of miR-155, single-molecule fluorescent in situ hybridisation (smFISH) was performed using a BaseScope duplex detection assay. See ESM Methods for additional details.

### Machine learning-based smFISH image analysis

Spatial quantification of pre-miRNA expression in single beta cells was composed of two steps: (1) smFISH image processing; and (2) machine learning-based classification. We followed the protocol described in our previous publication to process the smFISH images of pre-miRNA-155 in human pancreatic islets [24]. See ESM Methods for additional details.

### Recombinant adeno-associated virus production

A recombinant adeno-associated virus (rAAV) carrying mouse anti-miR-155 or a scrambled control (miR-Scr) was synthesised and produced as described previously [25]. Briefly, the HEK293F cell line was used for rAAV vector production. The cells were cultured in Viral Production Medium (Thermo, A4817901) in shaker flasks at 0.20 *g* at 37°C and 8% CO_2_. Cell viability was maintained at >95%, and transfection was performed when the cell density was at ~2.5×10^6^ cells/ml using the standard triple-plasmid transfection method of pRep2Cap8 (capsid 8), pHelper and pITR-AAV-miR-155 at a mol/l ratio of 1:1:1. These cells were collected at 72 h post-transfection and centrifuged at 3000 *g* for 15 min to separate the cells and media. The cells were resuspended in a lysis buffer of 50 mmol/l Tris-HCl (pH 8.2), put through three freeze–thaw cycles to release rAAV viruses, and then centrifuged at 3000 *g* for 20 min to collect the supernatant fraction. This fraction was combined with the cell media and treated with DNase I (5 U/ml) for 1 h at 37°C to digest contaminating DNAs to improve the quality of the crude lysate. rAAV viruses in this crude lysate were purified using LC with an affinity column (POROS AAVX CaptureSelect; Thermo, A36651).

### Animal studies

Eight-week-old female and male NOD/ShiLT mice were purchased from Jackson Laboratory and bred in-house at the Indiana University Laboratory Animal Research Center (LARC). To investigate the therapeutic potential of beta cell-specific knockdown of miR-155 in type 1 diabetes, we injected 4-week-old female NOD mice (Jackson Laboratory, JAX001976, RRID: IMSR_JAX:001976) with adeno-associated virus (AAV) containing shRNA for miR-155 driven by the rat insulin promoter or AAV8-RIP1-miRZip-Scr (~1×10^11^ viral genome (vg)/mouse) for control conditions. Mice were randomly assigned to treatment group, and AAV administration was performed at the same time of the day to minimise circadian variations. After 6 weeks of AAV administration, an IPGTT was performed to assess beta cell function. On the day of the IPGTT, mice were fasted for 6 h and then injected with glucose at a dose of 1 g/kg lean body mass. Blood glucose levels were measured at 0, 10, 20, 30, 60, 90 and 120 min using an AlphaTRAK 3 glucometer (Zoetis). A cohort of mice was euthanised at 12 weeks of age, and pancreatic tissues were harvested, fixed with 4% PFA, embedded with paraffin and sectioned to measure insulitis, as described previously [26]. smFISH was used to determine the beta cell-specific knockdown of miR-155-5p. For studies involving diabetes incidence, mice were followed up to 25 weeks of age, and diabetes incidence was quantified, with diabetes being defined as two consecutive blood glucose readings over 13.9 mmol/l. Mouse studies were conducted in accordance with Animal Research: Reporting of In Vivo Experiments (ARRIVE) guidelines under protocols approved by the Indiana University School of Medicine Animal Use Committee. Experiments were conducted only in female NOD mice, given the higher disease penetrance in female compared with male NOD mice. Blood glucose measurements to define diabetes onset were conducted by non-blinded members of the main research team. IPGTTs were performed by staff in the Indiana Diabetes Research Center Islet & Physiology Core who remained blinded to treatment group.

### Immunohistochemistry

Pancreatic tissues from 12-week-old female NOD mice that were administered AAV8-RIP1-miRZip-155 or AAV8-RIP1-miRZip-Scr were harvested and fixed with 4% PFA overnight at 4°C and embedded in paraffin as described in our previous publications [24]. See ESM Methods for additional details.

### Quantification and statistical analysis

To identify differentially expressed miRNAs, we performed paired sample analysis using the DESeq2 package in the R statistical program [27]. Because samples were sequenced in two batches, the batch was included as a covariate in the design formula and batch effects were accounted for when performing differential expression analysis using the DESeq2 package (ESM Fig. 1). miRNAs with fold change ≥1.5 and *p*<0.05 were considered as being differentially expressed in cytokine-treated islets compared with untreated islets. For qRT-PCR analysis from islets and EVs, we used Fishers exact test to identify significant differences between cytokine-treated and untreated samples. Unpaired *t* tests (Mann–Whitney test, non-parametric) were used for the analysis of biosensor data from islet-derived EV miRNAs with 95% CIs. A one-way ANOVA was used for the comparison of human plasma-derived EV miRNAs from healthy control individuals, individuals with AAb^+^, and individuals with recent-onset type 1 diabetes. Receiver operating characteristic (ROC) curve analysis was performed to evaluate the discriminatory performance of EV-associated miRNAs in distinguishing non-diabetic individuals, individuals with AAb^+^ and individuals with recent-onset type 1 diabetes. The discriminative performance of the LSPR platform was evaluated by computing bias-corrected AUC value, employing fivefold cross-validation and 10,000 bootstrap samples, implemented in nlpred package, an R package optimised for small sample size [28]. *p* values are represented as *p≤0.05, **p≤0.01 and ***p≤0.001.

## Results

### Isolation and characterisation of human islet-derived EVs

**Fig. 1:**
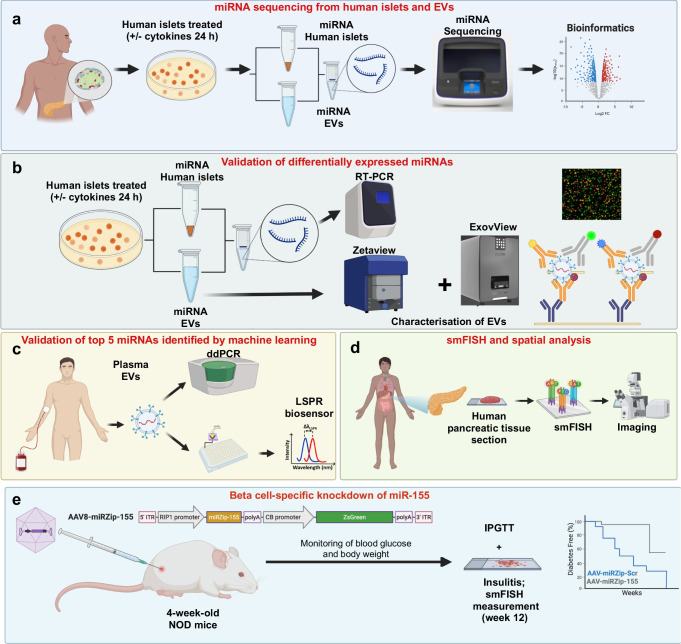
Schematic representation of the experimental workflow. Islets obtained from human cadaveric donors (*n*=10 total; 5 male/5 female) were treated with IL-1β + IFN-γ for 24 h. (**a**) Total RNA was isolated from islets and islet-derived EVs and subjected to small RNA sequencing. (**b**) Differentially expressed miRNAs were further validated using separate sets of islets and islet-derived EVs. (**c**) A panel of miRNAs selected from the top five differentially expressed EV miRNAs were validated using human circulating plasma-derived EVs from healthy control individuals, individuals with AAb^+^, and individuals with recent-onset type 1 diabetes. (**d**) miR-155 expression patterns were analysed in pancreatic samples from human organ donors with AAb^+^, donors with type 1 diabetes and non-diabetic control donors. (**e**) To determine the effect of miR-155 inhibition, 4-week-old female NOD mice were injected with AAV-miR-155 or AAV-miR-Scr. Glucose tolerance and insulitis were assessed after 8 weeks of injections (at 12 weeks of age). Created in BioRender. Syed, F. (2026) https://BioRender.com/omq3ztl. ddPCR, droplet digital PCR; FC, fold change; ITR, inverted terminal repeat; polyA, polyadenylation; ZsGreen, Zoan Green fluorescent protein

**Fig. 2:**
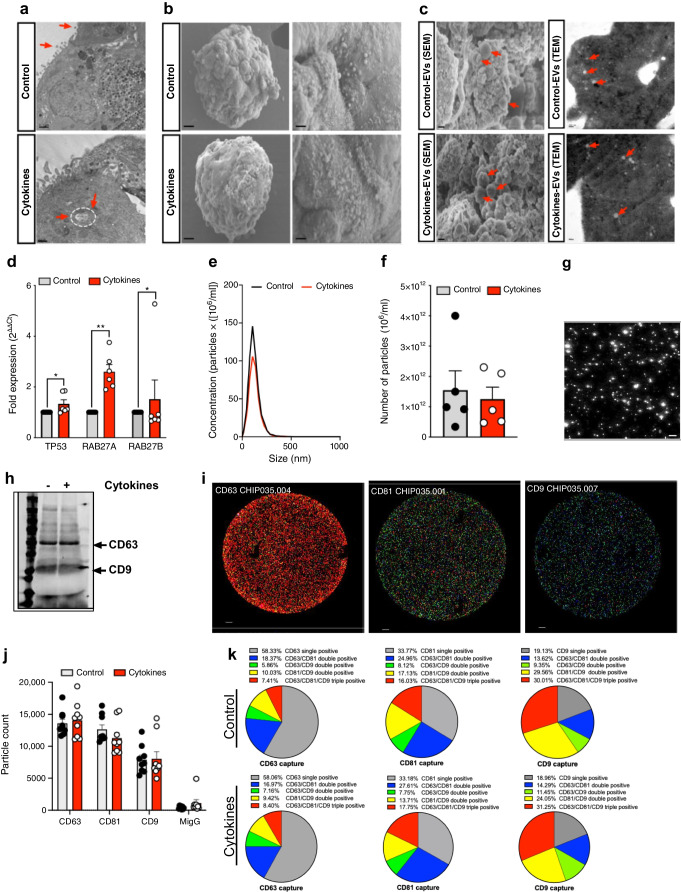
Characterisation of isolated EVs. Human islets were treated with or without 50 U/ml IL-1β and 1000 U/ml IFN-γ for 24 h. (**a**) Islets were subjected to TEM analysis to identify the presence of EVs over the islet plasma membrane and MVBs inside the cytoplasmic regions of pancreatic beta cells, indicated by red arrows. Scale bar, 0.5 µm. (**b**) SEM images of human islets show the presence of EVs over the islet plasma membrane. Scale bar, 100 µm. (**c**) Representative SEM and TEM images of isolated EVs from control and cytokine-treated islet culture supernatant fractions. Scale bar, 10.0 µm (SEM) or 500 nm (TEM). (**d**) qRT-PCR analysis of markers involved in EV biogenesis in control and cytokine-treated islets (*p≤0.05, **p≤0.01. *n*=6). (**e**, **f**) NTA showing size distribution graphs for isolated EVs from control (black line) and cytokine-treated (red line) islets. (**g**) Bright field images from NTA. Scale bar, 10 µm. (**h**) Immunoblot analysis for CD63 and CD9 from isolated EVs. (**i**) Images of ExoView showing the presence of EVs captured using CD63, CD81 and CD9; mouse IgG was used as a negative control. Scale bar, 10 µm. (**j**, **k**) Quantification of EV particles (**j**) and co-localisation of tetraspanin markers (CD63, CD81 and CD9) with each other indicated marker from control and cytokine-treated human islet culture supernatant fractions (**k**) (*n*=3, imaged at 3 different regions of interest). MigG, mouse IgG; RAB27A, Ras-related protein-27A; RAB27B, Ras-related protein-27B; TP53, tumour protein 53

The experimental workflow is shown in Fig. 1. EVs were isolated from human islet cultures treated with or without IL-1β + IFN-γ and characterised using SEM, TEM, NTA, qRT-PCR, immunoblot and single-particle interferometric reflected imaging of EV cargo. Human islets treated with or without cytokines showed similar ultrastructural morphology of intracellular multivesicular bodies (MVBs), as determined by TEM analysis (Fig. 2a), and SEM analysis confirmed the presence of EV particles in the plasma membrane (Fig. 2b). SEM and TEM analysis of EVs isolated from control and cytokine-treated human islet (*n*=3) conditioned media confirmed the presence of EVs, and similar ultrastructural features of EVs were noted under both conditions (Fig. 2c).

Cytokine treatment increased the expression of several transcripts involved in EV biogenesis, including *TP53, RAB27A,* and *RAB27B* (Fig. 2d; *n*=6). However, NTA (*n*=5) showed that human islet-derived EVs exhibited a uniform size range of 50–200 nm with no significant difference in particle concentration or size distribution between control or cytokine-treated human islets (Fig. 2e–g). Immunoblot analysis of isolated EVs confirmed the presence of the tetraspanin EV markers CD63 and CD9 (Fig. 2h). EVs were isolated (*n*=3) using dual size-exclusion chromatography and characterised using single-particle interferometric reflected imaging (Fig. 2i). Results from this analysis showed that a higher proportion of islet-derived EVs were positive for the classical EV-tetraspanin markers CD63 and CD81 compared with CD9 (Fig. 2j). However, sequential capture using CD63, CD81 and CD9 showed that the distribution of these markers across distinct EV populations was largely consistent between control and cytokine conditions (Fig. 2k).

### Inflammatory cytokine treatment leads to differential expression of miRNAs in human islets and islet-derived EVs

Next, RNA isolated from control and cytokine-treated islets (*n*=10) and islet-derived EVs (*n*=10) was subjected to unbiased small RNA-seq. A total of 83,141,746 and 87,979,932 reads were detected in samples obtained from control and cytokine-treated islets, respectively. Of these, ~65% of the reads in control islets and 67.5% of the reads in cytokine-treated islets aligned to the reference genome. Approximately one-third of the aligned reads (~32%) mapped to mature miRNAs in both control and cytokine-treated samples (ESM Table 3a). Similarly, from a total of 41,201,153 and 48,903,611 reads in samples obtained from EVs from control and cytokine-treated islets, respectively, ~58% and 53% of the total reads aligned to the reference genome. Approximately 28% of the aligned reads mapped to mature miRNAs in both experimental groups (6,588,292 reads in the control group and 8,416,500 reads in the treated group; ESM Table 3b). The samples were sequenced in two batches, and batch effects were accounted for when performing DESEq2 analysis (ESM Fig. 1).

**Fig. 3:**
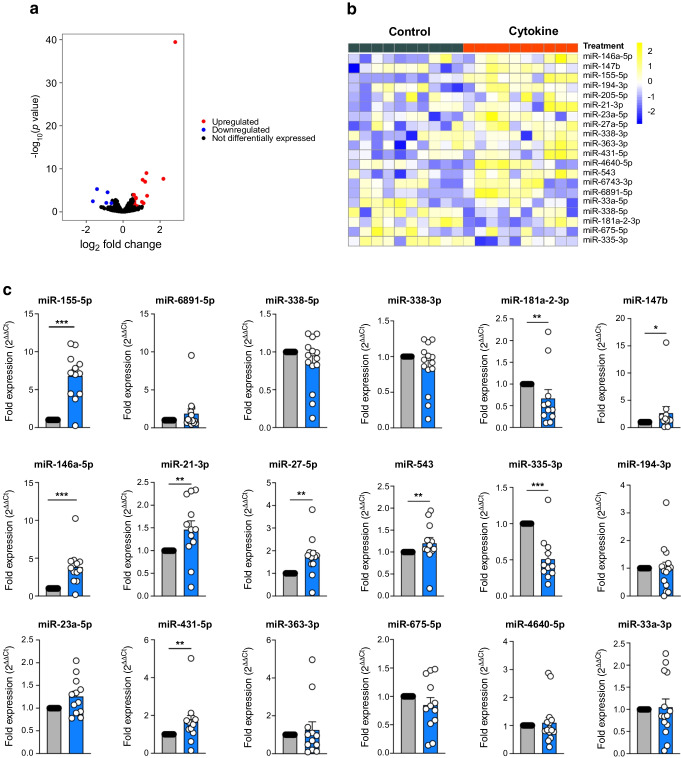
Identification and validation of differentially expressed islet-derived miRNAs. Human islets were treated with or without 50 U/ml IL-1β and 1000 U/ml IFN-γ for 24 h. (**a**) Volcano plot showing upregulated and downregulated miRNAs from human islets treated with or without cytokines. (**b**) Heatmap illustrates the expression of the 20 differentially expressed miRNAs identified from small RNA-seq analysis of control and cytokine-treated human islets (*n*=10; 5 male/5 female origin). The numbers on the scale (−2 to 2) represent scaled variance stabilising transformation (VSD) counts. (**c**) qRT-PCR validation of upregulated and downregulated miRNAs identified from sequencing analysis of human islets. Grey bars, control; blue bars, cytokine treatment. *n*=12 (7 male/5 female origin). *p≤0.05, **p≤0.01, ***≤0.001. Heatmap: blue indicates miRNAs with low read counts (low expression) and yellow indicates miRNAs with high read counts (upregulated, high expression)

**Fig. 4:**
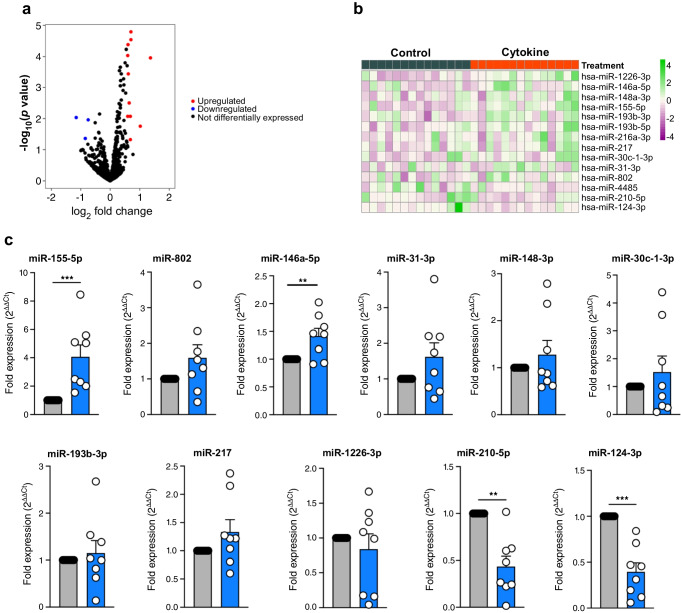
Identification and validation of differentially expressed islet-derived EV miRNAs. Human islets were treated with or without 50 U/ml and 1000 U/ml IFN-γ for 24 h, and EVs were isolated from the culture supernatant. (**a**) Volcano plot showing upregulated and downregulated EV miRNAs isolated from human islets treated with or without cytokines. (**b**) Heatmap illustrates the expression pattern of 14 differentially expressed EV miRNAs identified from small RNA sequencing analysis of isolated EVs from control and cytokine-treated islet culture supernatant fractions. (*n*=14; from 7 male/7 female origin). The numbers on the scale (−4 to 4) represent scaled variance stabilising transformation (VSD) counts. (**c**) qRT-PCR validation of upregulated and downregulated EV miRNAs identified from small RNA-seq analysis. Grey bars, control; blue bars, cytokine treatment. *n*=8 (from 4 male/4 female origin). **p≤0.01, ***p≤0.001. Heatmap: purple indicates miRNAs with low read counts (low expression) and green indicates miRNAs with high read counts (high expression)

Our methodology was robust, as 1110 and 890 miRNAs were retained in the datasets for control and cytokine-treated islets, respectively, after filtering for read counts. Yet, the evaluation was highly selective as a small, discrete subset of miRNAs were differentially expressed upon cytokine exposure. The volcano plot in Fig. 3a represents miRNAs that were significantly downregulated or upregulated in cytokine-treated islets compared with control islets. Twenty differentially expressed miRNAs were identified in islets, of which 15 were upregulated and five were downregulated by cytokine treatment (Fig. 3b and ESM Table 4). Similarly, the volcano plot in Fig. 4a depicts differentially expressed miRNAs identified from islet-derived EVs. Notably, we detected 14 differentially expressed miRNAs from EVs, 11 of which were upregulated and three of which were downregulated by cytokine treatment (Fig. 4b and ESM Table 4).

### Validation of differentially expressed miRNAs from human islets and islet-derived EVs

Next, we validated the differential expression of miRNAs identified in our sequencing data using human islets isolated from a separate set of human organ donors (*n*=8). As detailed above, islets were treated with or without cytokines (IL-1β and IFN-γ). For this analysis, RNA was isolated and miRNA expression levels were measured by qRT-PCR. Of the 20 differentially expressed miRNAs in islets (Fig. 3b), 19 were measured by qRT-PCR, and of the 14 differentially expressed miRNAs in EVs (Fig. 4b), 11 were measured by qRT-PCR. A total of four targets were omitted from qRT-PCR validation because of inability to find a suitable primer set. Targeted analysis validated changes in 18 islet miRNAs (13 upregulated and five downregulated; Fig. 3c) and 11 EV miRNAs (eight upregulated and three downregulated; Fig. 4c). Therefore, ~80% of the miRNAs identified in our small RNA sequencing showed similar expression patterns in qRT-PCR performed in a separate cohort of samples. However, only changes in a subset of seven upregulated and two downregulated islet miRNAs reached statistical significance (Fig. 3c). Analysis of EV miRNA expression patterns by qRT-PCR showed that two upregulated and two downregulated miRNAs were significantly modulated by cytokine treatment (Fig. 4c). Strikingly, results from small RNA-seq and qRT-PCR analysis indicated that only two miRNAs (miR-155-5p and miR-146a-5p) were commonly upregulated in both islets and EVs upon cytokine treatment.

Given the cellular heterogeneity of human islets, next we measured the expression of miR-155-5p and miR-146a-5p in EVs isolated from cytokine-treated EndoC-βH1 cells, a human beta cell line (ESM Fig. 2a, b). Similar to results observed in human islets, cytokine treatment induced upregulation of miR-155-5p and miR-146a-5p in EndoC-βH1-derived EVs, confirming that beta cells are one contributing source of these inflammatory miRNAs.

### Ranking of significant EV-derived miRNAs and functional enrichment analysis

**Fig. 5:**
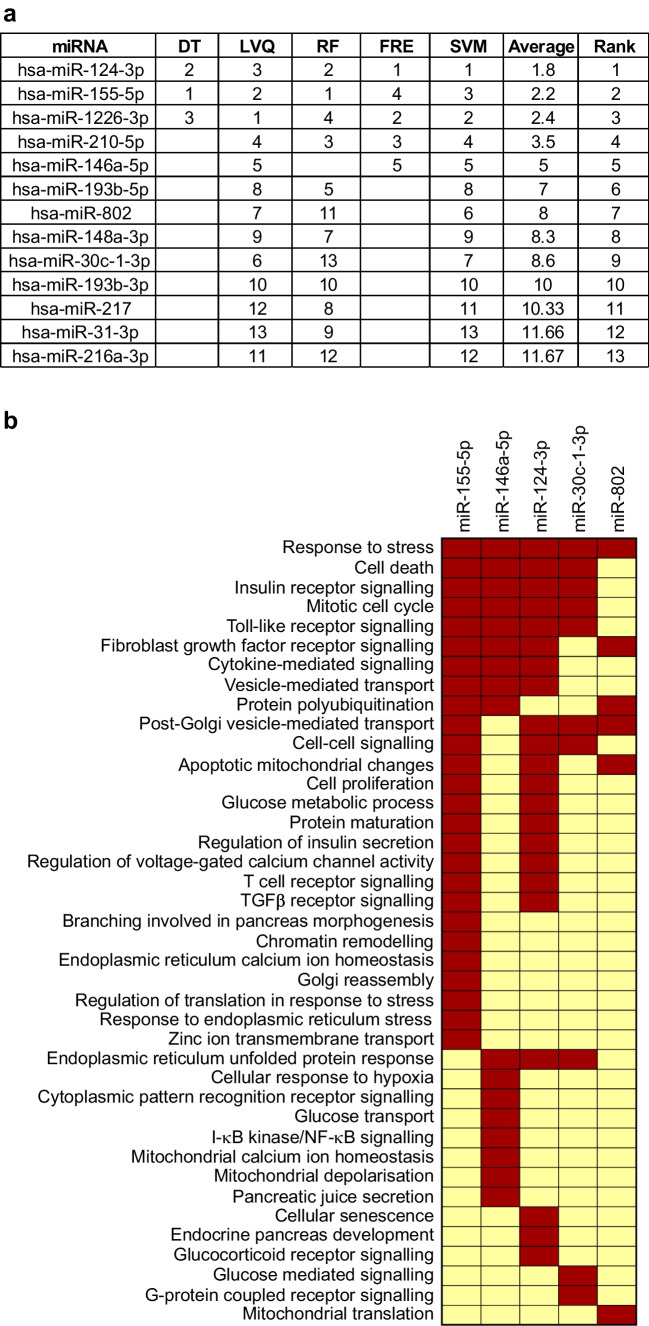
Differentially expressed miRNA rankings, miRNA-regulated biological functions and evaluation of miRNA expression in human islets. (**a**) Table of the top differentially expressed miRNAs from islet-derived EVs, evaluated using machine learning tools to generate coefficient values from the data that were used to rank the miRNAs according to signal performance. Lower values indicate better performance. (**b**) Predicted impact of miRNAs 155-5p, 146a-5p, 124-3p, 30c-1-3p, 802 and upon biological processes. Brown indicates enrichment of the pathway for a particular miRNA and yellow indicates absence of that pathway. DT, decision tree; FRE, feature reconstruction error; LVQ, learning vector quantification; RF, random forest; SVM, support vector machine

A major goal of our analysis was to define a signature of EV miRNAs with the potential to act as type 1 diabetes biomarkers. To rank the discriminatory ability of differentially expressed EV-derived miRNAs to distinguish between cytokine and control conditions, we applied multiple machine learning algorithms. This analysis identified ten miRNAs with the highest ranking based on coefficient values, where a lower coefficient value indicated better performance across all algorithms (Fig. 5a). Among these ten miRNAs, five (miR-155-5p, miR-146a-5p, miR-30c-1-3p, miR-802 and miR-124-3p) were advanced for analysis in a clinical cohort. The criteria applied to advance these miRNAs were based on a combination of previous literature linking them with beta cell dysfunction or autoimmunity, their rank in the integration of the machine learning analysis, and results from the qRT-PCR validation (Fig. 4c). Targets were predicted using Targetscan, microT-cds and Tarbase. Gene ontology terms, specifically terms associated with biological processes with *p*<0.05, were considered. The functional significance of these five miRNAs was identified using DIANA miRPath v3.0. Forty selected gene ontology terms are represented in Fig. 5b. The identified miRNAs were enriched for numerous processes associated with type 1 diabetes, including immune response, TLR signalling, inflammatory signalling, cell death and apoptosis.

### Validation of miRNA signatures in plasma samples of type 1 diabetes clinical cohorts

**Fig. 6:**
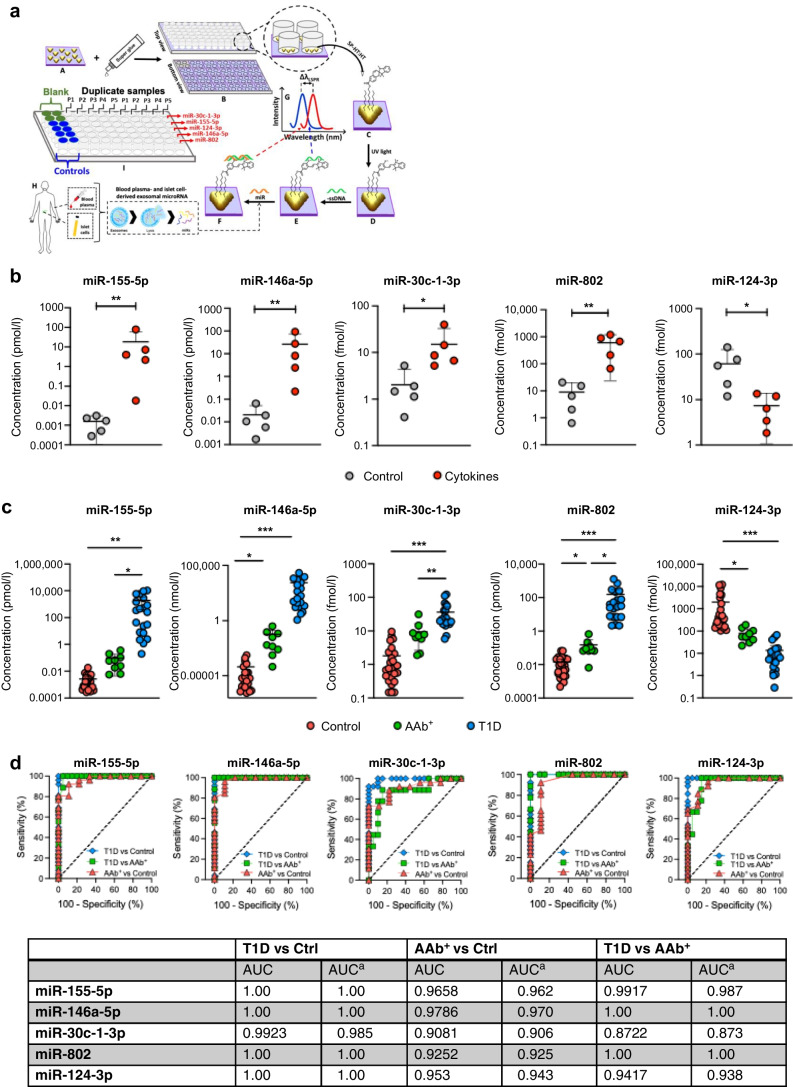
Quantification of miRNA expression patterns in plasma-derived EVs. (**a**, **b**) Fabrication and development of custom, label-free LSPR-based biosensors (**a**) and LSPR-based biosensor determination of miRNA expression levels in EVs isolated from control and cytokine-treated human islet culture supernatant fractions (**b**). (**c**) Circulating plasma EVs were isolated from individuals with AAb^+^ and recent-onset type 1 diabetes, and healthy control individuals. LSPR-based biosensor multi-plex miRNA assay results showing quantification of five miRNAs (155-5p, 146a-5p, 30c-1-3p, 802 and 124-3p) from human plasma-derived EVs. (**d**) ROC AUC analysis for measured miRNAs for the following comparisons: type 1 diabetes vs control; AAb^+^ vs control; and type 1 diabetes vs AAb^+^. AUC results and ^a^biased-reduced estimation of AUCs are shown in the table. Human islet-derived EVs, *n*=5; Human plasma-derived EVs, healthy controls, *n*=26 (17 male/9 female); AAb^+^, *n*=8 (5 male/3 female); new-onset type 1 diabetes, *n*=20 (11 male/9 female). *p≤0.05, **p≤0.01, ***p≤0.001. Ctrl, control; SP-HT-HT, spiropyran hexanethiol:hexanethiol spacer; T1D, type 1 diabetes

To test the utility of this signature of miRNAs to act as biomarkers capable of identifying type 1 diabetes risk, we isolated EVs from fasting plasma samples collected from individuals with AAb^+^ or recent-onset stage 3 type 1 diabetes, and from age-matched non-diabetic controls. The clinical characteristics of these individuals are shown in Table 1. We aimed to develop a platform that provided robust analytical accuracy, so we designed custom, label-free LSPR-based biosensors (Fig. 6a) to measure individual miRNA species. In brief, this method uses gold triangular nanoprisms that are functionalised with 75% spiropyran hexanethiol : 25% hexanethiol spacer (SP-HT:HT) to construct a biosensor that allows the measurement of a wide range of concentrations (e.g. nanomolar to attomolar ) with higher sensitivity and specificity compared with standard RT-PCR methods. This approach also uses a simple UV–visible spectrophotometer in the absorbance mode, making it potentially high-throughput for clinical settings [29–31] (ESM Fig. 3a–e).

**Table 1:** Clinical and anthropometric characteristics of human plasma donors

Disease status	Total no. of participants (*n* male/*n* female)	Age (mean ± SD)	BMI (mean ± SD)
Healthy (control)	26 (17/9)	11.38±4.02	19.46±5.10
AAb^+^	8 (5/3)	13.75±1.98	25.04±4.65
Recent-onset type 1 diabetes	20 (11/9)	12.80±3.38	19.13±4.30

First, we validated this approach by using the LSPR-based biosensors to measure expression levels of miR-155-5p, miR-146a-5p, miR-30c-1-3p, miR-802 and miR-124-3p in EVs isolated from control and cytokine-treated human islets (*n*=5) (Fig. 6b). The results of this analysis were concordant with results from qRT-PCR and RNA-seq. Next, we measured the plasma-derived EV miRNA signatures from the clinical cohort using LSPR biosensors. We observed a significant upregulation of miR-155-5p, miR-146a-5p, miR-30c-1-3p and miR-802, and decreased abundance of miR-124-3p, in plasma EVs from individuals with new-onset type 1 diabetes compared with non-diabetic control individuals (Fig. 6c). In addition, we observed upregulation of miR-146a-5p and miR-802 and downregulation of miR-124-3p in individuals with AAb^+^ (Fig. 6c) compared with non-diabetic control individuals. The sensitivity and specificity of this approach was robust, with ROC AUCs ranging from 1 to 0.8722 for between-group comparisons (Fig. 6c, d). These results remained robust when AUCs were adjusted using a bias-reduced cross-validated estimation approach (Fig. 6d). Consistent with previous results [29–32], the sensitivity and specificity of the LSPR biosensors were superior to droplet digital PCR (ddPCR), where the ddPCR ROC AUCs ranged from 0.5070 to 0.6560 for miR-155-5p and from 0.5089 to 0.7364 for miR-146-5p (ESM Fig. 2c–f).

### Localisation and spatial distribution of miR-155-5p in human pancreatic tissue sections using in situ hybridisation

**Fig. 7:**
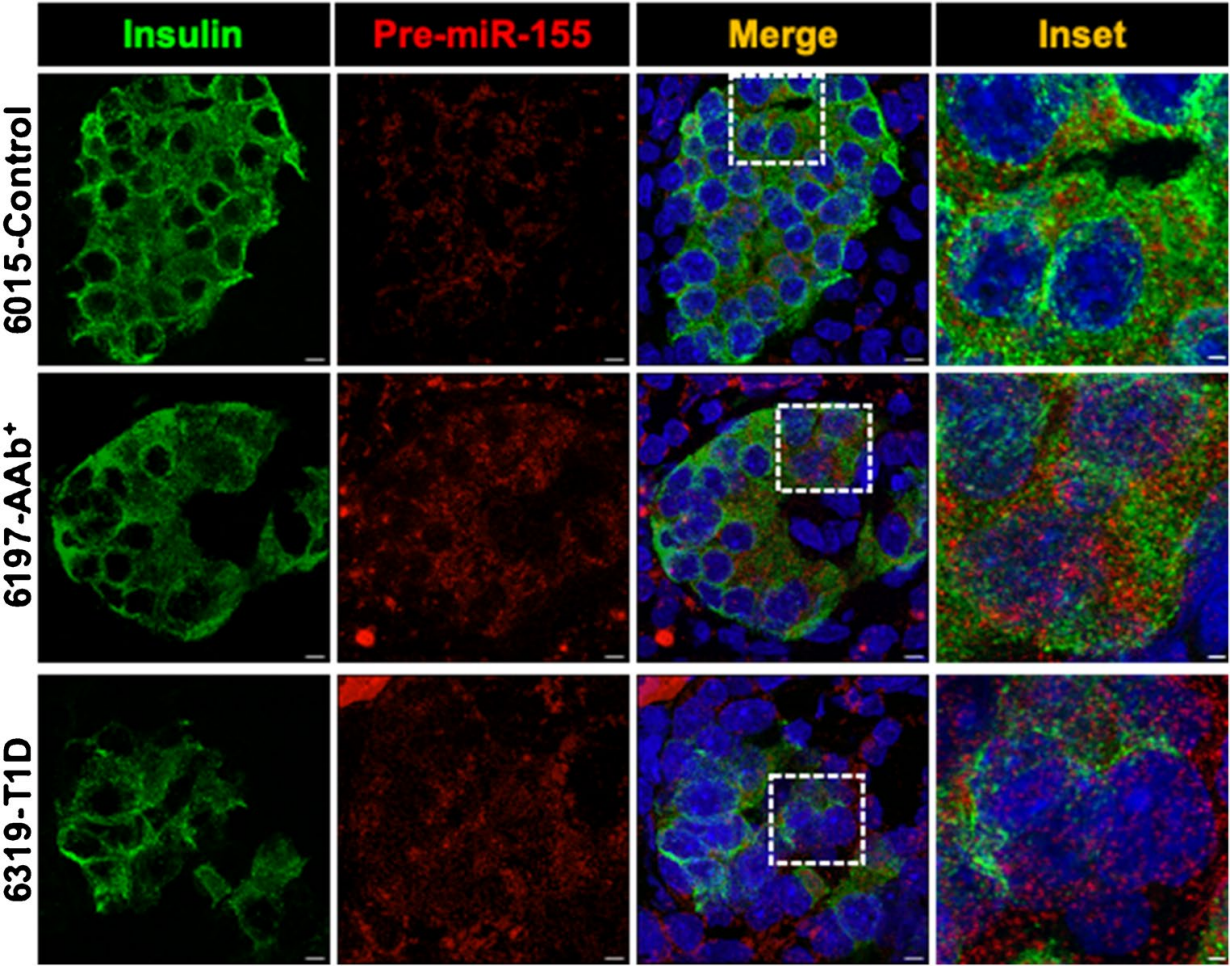
In situ hybridisation of pre-miRNA-155 expression in beta cells of human pancreas tissue sections. Representative smFISH images showing the expression of pre-miRNA-155 (red) in human pancreatic tissue sections obtained from non-diabetic control, AAb+, and type 1 diabetes human organ donors and counterstained with insulin (green) and nucleus (DAPI). Scale bar, 10 µm (inset, 1 µm)

While the miRNA content of EVs can inform biomarker strategies, intracellular miRNA expression and subcellular localisation are known to regulate a variety of molecular pathways in the beta cell, including those associated with type 1 diabetes pathophysiology [33]. Here, we focused on miR-155-5p for downstream validation as it was the most highly upregulated miRNA in our islet sequencing and qRT-PCR datasets (Fig. 3a, b). We performed smFISH imaging of pre-miR-155-5p on pancreatic tissue sections from human donors with AAb^+^ (*n*=2) and established type 1 diabetes (*n*=4), and non-diabetic control donors (*n*=4) by co-labelling with DAPI for the nucleus and anti-insulin antibody to identify beta cells (Fig. 7). ESM Fig. 4 shows negative and positive controls for our smFISH workflow.

**Fig. 8:**
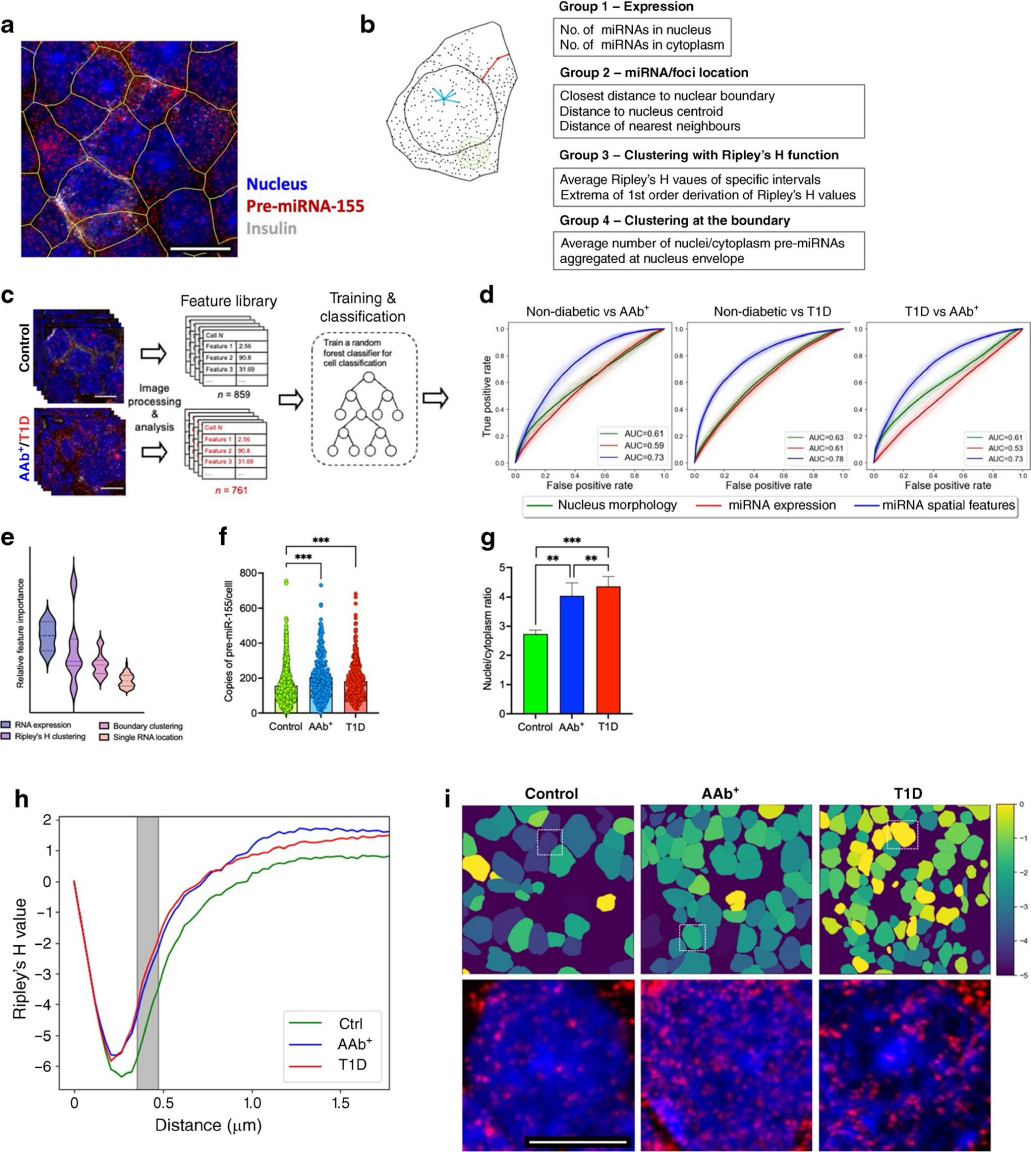
Machine learning-based spatial transcriptomics analysis of pre-miRNA-155 expression in beta cells. (**a**) Expression of the pre-miRNA-155 (red) was screened by smFISH imaging, with nuclei stained with DAPI (blue) and co-staining with insulin (grey). Cell outlines are depicted in yellow. Scale bar, 10 µm. (**b**) Quantitative features that describe the location of the pre-miRNA molecule in a single beta cell. Four groups of features were extracted for a single cell: pre-miRNA expression features (group 1); pre-miRNA location features (group 2); clustering features (group 3); and boundary clustering features (group 4). (**c**) Supervised machine learning was applied to three categories of smFISH image data (control, AAb^+^ and type 1 diabetes) for training and classification. (**d**) Single beta cells belonging to different categories (control, AAb^+^ and type 1 diabetes) were classified in a pairwise setting, the ROC curves were produced for each classifier, and the AUC was calculated for performance comparison. (**e**) Distinctive features were ranked to show the expression and clustering of the pre-miRNAs that dominate the classification. (**f**) Spatial expression of total pre-miRNA-155 in each beta cell was compared for all three groups (control, AAb^+^ and type 1 diabetes). (**g**) The comparison of the nucleus/cytoplasm ratio of pre-miRNA-155 in beta cells of control, AAb^+^ and type 1 diabetes samples. (**h**) Ripley’s H value at the distance of 457 nm (inset) for nuclear pre-miRNA-155 in a population of cells from all three categories (control, AAb^+^, and type 1 diabetes). (**i**) Cellular heatmap showing the Ripley’s H feature value for each cell; zoomed-in smFISH images of the insets from the heatmap are shown. Scale bar, 0.5 µm. **p≤0.01, ***p≤0.001. Ctrl, control; T1D, type 1 diabetes

Next, we developed an imaging-based bioinformatics approach and applied a supervised machine learning algorithm to our in situ hybridisation analyses. Imaging characteristics of miRNA molecules in a single beta cell were mathematically quantified using a series of features, including the expression level of the pre-miRNA in the nucleus or cytoplasm (group 1), the location of single miRNAs in the beta cell (group 2), the clustering of pre-miRNA-155-5p (group 3) and the aggregation of pre-miRNA-155-5p at the nuclear boundary (group 4) (Fig. 8a, b). The group 1 and 2 features of smFISH images have been successfully leveraged in other studies with different cell types [34]. However, these two feature groups were not adequate to differentiate non-diabetic samples from the type 1 diabetes samples with high accuracy. To this end, we integrated a mathematical model of spatial clustering using Ripley’s K function and its normalised derivative, Ripley’s H function [35, 36]) to characterise the degree of clustering for the miRNA and determine the aggregation of miRNA around the nuclear boundary.

The supervised machine learning algorithm was applied to 80% of the total cells (*N*=859 for non-diabetic samples, 307 for AAb^+^ samples and 454 for type 1 diabetes samples) for training of the classifier (Fig. 8c, d), while the remaining 20% of the total cells were used to evaluate the accuracy of the classifier. To fully assess the classification performance of pre-miRNA spatial features, we conducted pairwise classification between non-diabetic, AAb^+^ and type 1 diabetes samples using the classifier trained with pre-miRNA spatial features. This classifier was compared with classifiers trained with pre-miRNA expression alone and classifiers trained with nucleus morphology. The area under the ROC curve indicated that the spatial feature-trained classifiers consistently outperformed the classifiers trained with pre-miRNA expression and nucleus morphology in all pairwise classifications (Fig. 8c, d).

Interestingly, the spatial expression of the pre-miRNA and the local clustering effects dominated the classification process (Fig. 8e). In addition, spatial analysis of pre-miR-155 expression from insulin-positive beta cells showed an increase in pre-miR-155 copy number from individuals with AAb^+^ and type 1 diabetes vs control individuals (Fig. 8f). Moreover, subcellular localisation of pre-miR-155 showed a spatial disparity in pre-miR-155 expression, where we observed an increase in the nuclear to cytoplasmic ratio in the samples from individuals with AAb^+^ and type 1 diabetes samples compared with control donors (Fig. 8g). In line with this data, the colour-coded cell images based on the Ripley’s H function at 457 nm suggested a different degree of pre-miRNA-155 clustering in tissue sections from control individuals and those with AAb^+^ and type 1 diabetes (Fig. 8h), and this was clearly observed in the raw smFISH images (Fig. 8i). Our findings are the first to indicate that the physical distribution of pre-miRNA may be directly associated with beta cell stress and dysfunction.

### Beta cell-targeted inhibition of miR-155-5p in prediabetic NOD mice improves glucose tolerance and reduces insulitis

**Fig. 9:**
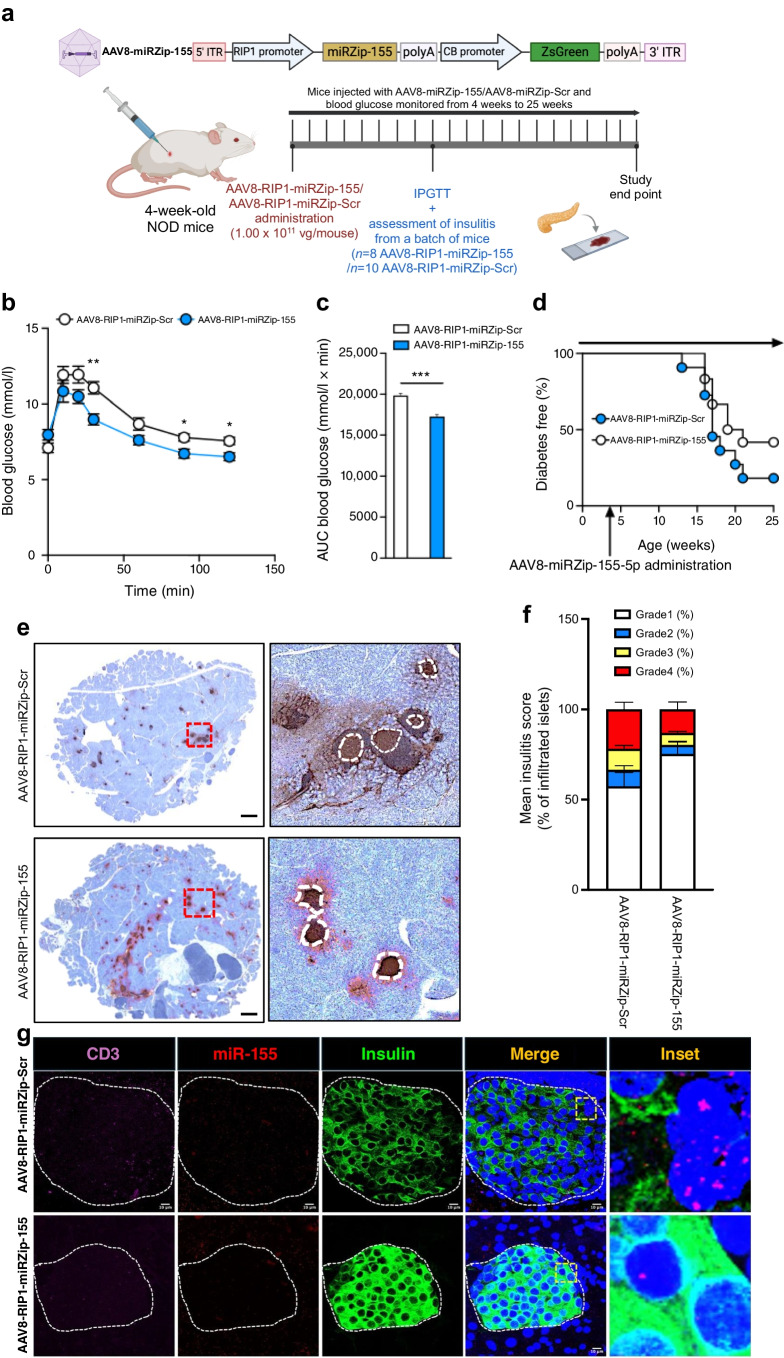
In vivo effect of beta cell-targeted inhibition of miR-155-5p by AAV8-RIP1-miRZip-155-5p in prediabetic NOD mouse models. (**a**) Schematic of beta cell-specific knockdown of miR-155 using an AAV8-RIP1-miRZip-155 construct in 4-week-old NOD mice. Created in BioRender, Syed, F. (2026) https://BioRender.com/lhqsav0. (**b**) IPGTT from 12-week-old NOD mice treated with AAV8-RIP1-miRZip-155 (blue circles) or AAV8-RIP1-miRZip-Scr (white circles). (**c**) AUC of IPGTT data. *n*=10 AAV8-RIP1-miRZip-Scr, *n*=15 AAV8-RIP1-miRZip-155. (**d**) Kaplan–Meier survival plot showing diabetes incidence in the two groups. (**e**) Immunohistochemistry images of insulin staining in pancreatic tissue sections from AAV-treated mice. Representative images illustrating reduced immune cell infiltration in islets of mice treated with AAV8-RIP1-miRZip-155 compared to islets of mice treated with AAV8-RIP1-miRZip-Scr. Areas shown in dotted red squares indicate zoomed regions of interest. Insulin positive regions marked in white circles. (**f**) Quantification of the mean insulitis scores and distribution of insulitis grade between interventions. (**g**) smFISH analysis of pancreatic tissue sections showing expression of miR-155 (red) and *Cd3e* mRNA (magenta) in pancreatic tissues of mice treated with AA8-RIP1-miR-155-5p or AA8-RIP1-miR-Scr mice. Tisses were counterstained for insulin protein (green), and nuclei were identified with DAPI staining. Scale bar, 200 µm (**e**) or 10 µm (**g**). ITR, inverted terminal repeat; polyA, polyadenylation; vg, viral genome; ZsGreen, Zoan Green fluorescent protein

Finally, to understand the impact of miR-155 inhibition in beta cells in vivo, we generated an AAV construct containing anti-miR-155 driven by the rat insulin promoter 1 (RIP1), AAV-RIP1-miR-155, to specifically target beta cells (Fig. 9a). AAV-RIP1-miR-155 or AAV-miR-scrambled (Scr) was administered to 4-week-old female NOD mice via i.p. injection. After 8 weeks, the mice were subjected to an IPGTT during which the mice administered AAV-RIP1-miR-155 (*n*=15) had lower glucose excursions (Fig. 9b), with a significant reduction in the glucose AUC compared with mice injected with AAV-RIP1-miR-Scr (*n*=10) (Fig. 9c). A small cohort of AAV-RIP1-miR-155- (*n*=12) or AAV-RIP1-miR-Scr-injected mice (*n*=10) were followed up to 25 weeks of age and monitored for diabetes incidence. A small, non-significant, difference in diabetes incidence was observed between mice treated with AAV-RIP1-miR-155 and those treated with AAV-RIP1-miR-Scr (Fig. 9d). Immunohistochemistry analysis of pancreatic tissue sections from AAV-RIP1-miR-155- (*n*=8) and AAV-RIP1-miR-Scr-treated mice (*n*=10) showed a significant reduction in insulitis when mice were injected with AAV-RIP1-miR-155 (Fig. 9e, f). In addition, smFISH analysis of pancreatic tissue sections showed reduced expression of miR-155 and *Cd3e* mRNA in pancreatic tissues of mice treated with AAV8-RIP1-miRZip-155 compared with mice treated with AA8-RIP1-miRZip-Scr (Fig. 9g). Together, these data suggest that beta cell-specific blockade of miR-155 expression may have therapeutic potential in reducing dysglycaemia and insulitis during type 1 diabetes pathophysiology, although the impact on diabetes incidence will need to be tested in a larger cohort of mice.

## Discussion

In this study, we aimed to identify islet miRNAs that may play a role in type 1 diabetes pathogenesis and define miRNA signatures of islet-derived EVs that could be leveraged as biomarkers to stratify type 1 diabetes risk. To this end, we used small RNA-seq to identify miRNAs that were differentially expressed in both human islets and islet-derived EVs using an in vitro and organ-based model of type 1 diabetes. Next, we validated a subset of these differentially expressed miRNAs in plasma from individuals with AAb^+^, individuals with recent-onset stage 3 type 1 diabetes, and age-matched non-diabetic control individuals. Finally, we developed a mouse model to demonstrate that inhibition of miR-155 improved glycaemic variables and reduced insulitis in NOD mice.

Several reports have shown that components of EV cargo, such as nucleic acids (DNA and RNAs), lipids and proteins, are dynamically modulated in response to extrinsic and intrinsic cues and may reflect the cellular physiological or pathological state of a given cell type [37, 38]. Moreover, EVs are protected from RNase-mediated degradation [39]. Hence, EV-derived miRNA signatures may serve as reliable markers for identifying the health status of a cell. Methods to identify indicators of beta cell stress can help dissect heterogeneity among at-risk individuals and could inform both the timing and selection of disease-modifying therapies. Current methods to do this in type 1 diabetes are lacking.

Recently, we used total RNA-seq to show that several types of RNA species, such as miRNAs, piRNAs, snoRNA, tRNAs and poly(A) RNAs, are packaged and secreted through human islet-derived EVs [40]. However, the number of miRNAs identified by this approach was low. To address this lack of enrichment, here we used a small RNA-seq approach in human islets and islet-derived EVs. This technique was robust, as we confidently identified 1110 miRNAs in islets and 890 miRNAs in EVs, which approached detection of ~50% of the 2000 known human miRNAs. Interestingly, a much smaller subset of these miRNAs was differentially expressed between control and cytokine treatment (20 in islets and 14 in EVs), suggesting that miRNA biogenesis under stress conditions is engaged with a high degree of selectivity. Recent studies have shed light on the regulation of tissue retention and sorting of miRNAs into EVs. It has become clear that this process is highly regulated and depends on the specific sequence motifs carried by the miRNAs [41]. For instance, Liu et al demonstrated that the sorting of miRNAs into EVs may occur through a process of liquid–liquid phase separation involving an RNA binding protein called YBX1 [42]. These findings have important implications for the use of EV-associated miRNAs as biomarkers, as the sorting process may affect the composition and abundance of miRNAs within EVs. In addition, understanding the mechanisms of miRNA sorting may lead to the development of new strategies for selectively loading miRNAs into EVs for therapeutic purposes. Further research is needed to fully elucidate the regulation of miRNA sorting into EVs and its implications for EV-based therapies and biomarker development.

Next, we applied a machine learning approach and multiple algorithms to prioritise a signature of miRNAs for clinical testing. Based on results from the machine learning analysis and validation of differentially expressed miRNAs using targeted qRT-PCR, we advanced miR-155-5p, miR-146a-5p, miR-30c-1-3p, miR-802 and miR-124-3p for analysis in plasma EVs isolated from children with AAb^+^ or recent-onset type 1 diabetes, and healthy control children. This signature was also informed by published literature linking these miRNAs with the following processes: inflammation and autoimmunity (miR-155-5p, miR-146a-5p and miR-124-3p) [43–45]; acinar–ductal trans-differentiation in the pancreas (miR-802) [46]; glucose-stimulated insulin secretion (miR124-3p) [47, 48]; and fibrosis of the pancreas (miR-30c-1-3p) [49]. We developed an LSPR-based biosensing approach to analyse all five selected miRNAs in human plasma-derived EV samples. Our results from the LSPR biosensor approach provided a significant dynamic range and improved discriminatory ability across groups compared with qRT-PCR. This approach is also quite robust as it does not require cDNA synthesis or PCR amplification steps, the biosensor works similarly to a microarray and is more amenable to the type of high-throughput analysis required in a clinical study. Using this platform, we observed a significant upregulation of miR-155-5p, miR-146a-5p, miR-30c-1-3p and miR-802-3p and a decreased abundance of miR-124-3p in plasma EVs from individuals with new-onset type 1 diabetes compared with non-diabetic control individuals. In addition, miR-146a-5p and miR-802 were increased and miR-124-3p was decreased in plasma EVs from individuals with AAb^+^ compared with control individuals, suggesting that these miRNA signatures could reflect a phenomenon of both early and late beta cell stress during type 1 diabetes development.

In line with our data, a recent study by Januszewski et al reported the association of 50 circulating plasma miRNAs with C-peptide levels in a cohort of individuals with and without type 1 diabetes. They identified 16 miRNAs, including miR-146a and miR-155, that showed a strong association with plasma C-peptide levels [50]. Furthermore, this group found that 13 miRNAs, including miR-155 and miR-210, were strongly associated with detectable C-peptide in individuals with type 1 diabetes [50]. Interestingly, Margaritis et al performed a meta-analysis using published studies in type 1 diabetes cohorts and identified seven miRNAs, including miR-181 and miR-210, that were significantly upregulated in individuals with type 1 diabetes [51]. Consistent with these data, we identified upregulation of miR-146a and miR-155 in islets and islet-derived EVs, and we found increased levels of these miRNAs in our clinical cohort. In contrast, we observed a downregulation of miR-210 in islet-derived EVs, suggesting that the source of miR-210 observed in other studies may emanate from another tissue type.

Finally, we aimed to link a promising biomarker candidate with type 1 diabetes pathogenesis. Here, we tested whether miR-155, the most highly upregulated miRNA across our datasets, was modulated similarly in human pancreases collected from individuals with AAb^+^ and individuals with type 1 diabetes. miR-155 was a compelling target for translation based on an abundance of previous literature linking it with type 1 diabetes pathogenesis. Notably, miR-155 promotes the secretion of type I IFN by dendritic cells [52], stimulates an M1 phenotype in macrophages within the islet milieu [53, 54], and plays a role in T cell activation [55], although its role in the beta cell has been less well studied.

We found that pre-miR-155-5p expression was increased in beta cells from donors with AAb^+^ and type 1 diabetes compared with non-diabetic donors. We aimed also to investigate the spatial localisation of miRNAs within beta cells of at-risk (AAb^+^) individuals and individuals with early-onset type 1 diabetes. Our results revealed that pre-miR-155-5p exhibits distinctive features of disease-predictive localisation, including increased nuclear localisation and clustering of pre-miRNA signals. We utilised the high sensitivity of the smFISH methodology along with machine learning algorithms for data analysis. The increased nuclear ratio of pre-miR-155 could be attributed to several possible mechanisms, such as nuclear processing of pre-miRNAs or nuclear import of processed miRNA from the cytosol through importin or nuclear pore complexes. Moreover, nuclear localisation of pre-miR-155 could signify an increase in non-canonical activities of the miRNA, such as regulation of transcription via target gene promoters and *cis*-regulatory elements (CREs), regulation of chromatin structure (looping), and other epigenetic changes such as methylation, compaction of chromatin and genomic remodelling. The self-clustering of miR-155 is suggestive of association with membrane-bound organelles or the formation of scaffolding complexes and RNA condensates [33, 56].

A recent study by Guay et al demonstrated that transfer of T lymphocyte-derived EV miRNAs, including miR-142-3p, miR-142-5p and miR-155, induced beta cell apoptosis whereas inhibition of these miRNAs in T lymphocytes significantly reduced type 1 diabetes incidence in NOD mice [17]. Our data extend these findings by showing that beta cell-specific inhibition of miR-155 also confers functional benefit. Pathway analysis from small RNA-seq indicates that miR-155 targets are enriched in pathways governing ER stress, mitochondrial dysfunction, calcium homeostasis and translation, suggesting that miR-155 could amplify beta cell stress responses linked with increased beta cell immunogenicity through neoantigen production, MHC class II upregulation and chemokine production [55, 57–59]. Consistent with this framework, prediabetic female NOD mice treated with AAV-RIP1-miR-155 exhibited improved glucose tolerance and reduced insulitis compared with mice injected with AAV-RIP1-miR-Scr. Although larger cohorts of mice are needed to conclude definitively whether this approach has a beneficial effect on type 1 diabetes development, our findings, in combination with those of Guay et al, suggest that targeting of miR-155 may provide complementary benefits in both the immune and endocrine compartments.

There are limitations of our study that should be noted. First, the sample size for the machine learning analysis to generate the EV miRNA signature was small. Typically, these algorithms perform most robustly with larger sample size and independent cohorts for both testing and validation. Despite this limitation, the predicted miRNAs performed well in distinguishing individuals with type 1 diabetes and those with AAb^+^ from non-diabetic control individuals in clinical testing. Additional experiments should be performed in AAb^+^ cohorts followed longitudinally before and after seroconversion and through type 1 diabetes development. Likewise, further analyses and additional experiments will be needed to determine the impact of additional demographic features such as age and sex on EV miRNA signatures. It is important to note that both human islets and plasma-derived EVs contain a heterogeneous mix of EVs from different cells and organs, respectively. While we identified our candidate miRNAs from an islet-based stress model, we cannot presume they provide information solely about the health status of the beta cell or that miRNAs measured in circulation emanate from the beta cell. We confirmed upregulation of miR-155-5p and 146a-5p in cytokine-treated EndoC-βH1 cells, validating the beta cell as at least one contributing source. However, it is possible that circulating levels of these target miRNAs could be modulated in other inflammatory and/or autoimmune conditions. Whether this finding would diminish their utility as a biomarker for type 1 diabetes risk will require further testing.

Notwithstanding these limitations, we identified a robust panel of miRNAs that were modulated in individuals with type 1 diabetes or AAb^+^, and we developed a novel plate-based analytical platform to measure miRNA targets. Strikingly, the expression patterns observed in our cross-sectional cohorts suggest that miRNA expression levels may be modulated progressively during the evolution of type 1 diabetes, suggesting that they may provide stage-specific information as potential biomarkers. Taken together, our results show that miRNA expression patterns in human pancreatic islets and islet-derived EVs change dynamically under inflammatory conditions and suggest that organ-based disease models in combination with label-free LSPR-based biosensors can be leveraged to inform biomarker and therapeutic strategies for type 1 diabetes.

## Supplementary Information

Below is the link to the electronic supplementary material.

ESM (PDF 1384 KB)

ESM Table 1 (XLSX 24 KB)

## Data Availability

The data from small RNA-seq of human islets and islet-derived EVs were deposited in GEO database (accession no. GSE160391). All the other data associated with this manuscript can be found in the manuscript or in the ESM files.

## Abbreviations

AAb: Diabetes-associated autoantibody
AAV: Adeno-associated virus
ddPCR: Droplet digital PCR
ER: Endoplasmic reticulum
EV: Extracellular vesicles
LSPR: Localised surface plasmon resonance
MVB: Multivesicular body
NTA: Nanoparticle tracking analysis
rAAV: Recombinant adeno-associated virus
RIP1: Rat insulin promoter 1
ROC: Receiver operating characteristic
SEM: Scanning electron microscopy
smFISH: Single-molecule fluorescent in situ hybridisation
TEM: Transmission electron microscopy
