# Supplementary material for: Beta cell microRNAs function as molecular hubs of type 1 diabetes pathogenesis and as biomarkers of diabetes risk
Source: Diabetologia. 2026 Apr 21;69(7):1975–96. doi: 10.1007/s00125-026-06720-7 (PMC13236762; doi:10.1007/s00125-026-06720-7)
Supplement: Supplementary file 1 — ESM (PDF 1384 KB) [file 125_2026_6720_MOESM1_ESM.pdf]

Electronic Supplementary Material

Beta Cell microRNAs Function as Molecular Hubs of Type 1 Diabetes Pathogenesis and as Biomarkers of Diabetes Risk

Authors: Farooq Syed[1,2,3,12,13*], Preethi Krishnan[3,4], Garrick Chang[5], Jyoti Rana[12], Sarah R. Langlais[6], Sumon Hati[6], Kentaro Yamada[1], Anh K. Lam[1], Sayali Talware[7], Jacqueline Del Carmen Aquino[2], Eli Hagedorn[1,2,3], Xiaowen Liu[8], Rajesh Sardar[6], Jing Liu[9], Raghavendra G. Mirmira[10], and Carmella Evans-Molina[1,2,3,4,11*]

Affiliations: [1]Department of Pediatrics, [2]Center for Diabetes and Metabolic Diseases, [3]Herman B Wells Center for Pediatric Research, and [4]Department of Medicine, Indiana University School of Medicine, Indianapolis, IN 46202, USA. [5]Department of Physics, [6]Department of Chemistry and Chemical Biology, and [7]School for Informatics and Computer, Indiana University Indianapolis, Indianapolis, IN 46202, USA. [8]Deming Department of Medicine, Tulane University School of Medicine, New Orleans, LA 70112, USA. [9]Department of Physics and Astronomy, Purdue University, West Lafayette, IN 47907. [10]Kovler Diabetes Center, University of Chicago, Chicago, IL 60637, USA. [11]Roudebush VA Medical Center, Indianapolis, IN 46202, USA. [12]Department of Diabetes-Immunology, Arthur Riggs Diabetes & Metabolism Research Institute, City of Hope, Duarte, CA, USA. [13]Center for RNA Biology and Therapeutics, Beckman Research Institute, City of Hope, Duarte, CA, USA

*Address correspondence to: cevansmo@iu.edu or fsyed@coh.org

LIST OF ESM MATERIALS

ESM Methods

ESM Table 1: Human islet checklist

ESM Table 2: microRNA sequences that were used to generate calibration curves

ESM Table 3: Descriptive statistics of islets and EVs

ESM Table 4: Differentially expressed miRNAs in islets and EVs

ESM Table 5: Single stranded DNA (*-ssDNA)* oligomer sequences used in this study (cited in ESM Materials only)

ESM Table 6: λ_{LSPR} responses of spiropyran based plasmonic nanosensors for microRNAs (cited in ESM Materials only)

ESM Table 7: λ_{LSPR} responses of spiropyran based plasmonic nanosensors for nucleic acid ss-DNA (cited in ESM Materials only)

ESM Fig. 1: Principal component analysis on islets and EVs

ESM Fig 2: Droplet digital PCR (ddPCR) analysis of miRNA expression of EV derived miRNAs from human plasma samples and EVs isolated from EndoC βH1 cells.

ESM Fig. 3: Calibration curve generated using synthetic microRNAs

ESM Fig 4: Representative images showing positive and negative controls of smFISH analysis

ESM Fig. 5: UV-Visible absorption spectra and scanning electron microscope image of Au TNPs (cited in ESM Materials only)

ESM Fig. 6: Representative UV-Vis extinction spectra showing Au TNP, SP-HT:HT, MC-HT:HT, -ssDNA-155-5p attachment and microRNA-155-5p hybridisation (cited in ESM Materials only)

ESM METHODS:

SEM and TEM imaging analysis of islets and EVs

Scanning electron microscopy (SEM) and transmission electron microscopy (TEM) imaging were performed at the Northwestern University Atomic and Nanoscale Characterization Experimental Center (NUANC) in Evanston, Illinois. After the indicated treatments, islets were fixed in freshly prepared buffer containing 2.5% glutaraldehyde, 2% paraformaldehyde, and cacodylate buffered fixative and then stored at 4°C. On the following day, the samples were exchanged into fresh fixatives. The samples were microwave processed in a Pelco Biowave built-in with a cold spot and vacuum to enhance sample processing. Samples were washed thrice with cacodylate buffer and incubated in a solution containing 1% OsO4 and 0.8% $KFeCN_6$. Subsequently, the samples were washed twice with cacodylate buffer and two washes with diH2O. The samples were then transferred to a solution containing 1% uranyl acetate (UA) in diH2O and washed thrice with diH2O. Samples were dehydrated twice with a series of different ethanol concentrations: 30%, 50%, 70%, 90%, and 100%. During the 100% ethanol step, the samples were divided and designated for SEM- or TEM-specific processing. For SEM imaging, the samples were dried using a Tousimis Samdri-795 Semi-Automated Critical Point Dryer. Dried samples were transferred into a SEM stub and coated with Pt/Pd. Imaging was performed on a Hitachi SU8030 SEM using an accelerating 2 or 5 kV voltage. Samples for TEM imaging were processed in a transitional fluid of acetone infiltrated with EMBed812 resin and polymerized at 60°C for 24 hours. Ultrathin sections were cut using a Diatome diamond knife using a Leica Ultracuts ultramicrotome. Cross-sections with a thickness of 90 nm were made and collected onto copper grids and imaged in a JEOL 1230 TEM with an 80 kV accelerating voltage using a Gatan Orius camera.

Microarray chip-based tetraspanin staining

Antibody coated microarray chips were incubated at room temperature for 15 min and pre-scanned using the ExoView R200 automated imaging system (NanoView Bioscience, USA). Chips were transferred to 24 well plates using tweezers, and EVs were isolated from control and cytokine-treated human islet culture supernatant using an in-house developed dual chromatography-based approach. Isolated EVs were diluted (1:10) using 1X sample incubation buffer, and 50 µL of the diluted sample was added to the chip and incubated for 16-24 hours at room temperature. After the incubation period, the chips in 24 well plates were transferred to an automated plate washer and washed thrice with 1000 µL of solution A, then 750 µL of buffer was removed and incubated with 250 µL fluorescently labeled capture antibodies: anti-CD9 (CF®488A), anti-CD81 (CF®555), and anti-CD63 (CF®647) at 1:500 dilution in blocking solution II and incubated for 1 hour in an orbital shaker at 8 x *g*. Next, the chips were washed thrice with 1X solution A, thrice with 1X solution B, and then thrice with DI water. The chips were placed at 45° angle, slowly pulling the chip out of the water, and placed on absorbent paper and protected from light. The chips were imaged using an ExoView R200 automated imager, and the data were analysed using ExoView data analysis software.

miRNA sequence data analysis

The raw sequencing files were aligned to the human genome (hg19) using STAR aligner v2.5.3a. Mature miRNAs were annotated using miRBase v20. miRNAs that had a total of less than 10 read counts were removed from further analysis. Read alignment, annotation, and generation of raw counts were completed using Partek Flow v6.0.18.0110 (Copyright ©; 2018 Partek Inc., St.

Louis, MO, USA). Paired sample analysis was used to identify DE miRNAs using the DESeq2 package in the R statistical program (1). miRNAs with fold change (FC) ≥ 1.5 and $p < 0.05$ were considered as DE in cytokine-treated islets (cases) compared to untreated islets (controls).

Selection of internal reference genes for quantitative RT-PCR for miRNAs

Potential reference miRNAs capable of serving as normalisation strategies for qRT-PCR were identified using sequencing data from human islets and islet-derived EVs. A molecule was considered a potential reference gene if expressed in high amounts in both cases and controls; therefore, only miRNAs with at least 10 read counts in all the samples were considered. Further, any miRNA that showed a statistical significance of $p < 0.1$ between cases and controls was removed. To assess the relative stability of miRNAs, we calculated the co-efficient of variations (CVs), and miRNAs with a CV < 0.3 were retained. In addition, miRNAs falling within the 10th percentile and showing a normal distribution with normality p-value > 0.6 (Shapiro-Wilk test) were identified. As a final filtering criterion, miRNAs with a fold change < 1.05 were considered potential reference genes. miRNAs identified from islets and islet-derived EVs were tested using qRT-PCR. RefFinder (http://150.216.56.64/referencegene.php#) was used to determine the most stable miRNA as it compares to the relative stability across different methods such as delta Ct, BestKeeper, NormFinder, and GeNorm, and gives a comprehensive ranking of the input RNAs.

cDNA synthesis and quantitative RT-PCR for mRNA and miRNAs

To determine the expression of genes involved in EV biogenesis, 0.5-1 μg of total RNA was isolated from human islets and reverse transcribed using an M-MLV RT kit (Invitrogen, MA,

USA). TaqMan primers (Applied Biosystems, CA, USA) were used for qRT-PCR. β-actin was used as a normaliser to determine the expression of TP53, RAB27A, and RAB27B. The data were presented as fold expression using the $2^{-\Delta\Delta}$Ct method compared to the untreated control.

For validation of DE miRNAs from human islets and islet-derived EV miRNAs, 1 µg of RNA isolated from human islets or 10 µL of EV RNA was reverse transcribed using the miRScript II kit (Qiagen, Germany), following manufacturer's protocol. qRT-PCR was performed using the miScript SYBR Green based PCR kit (Qiagen, Germany). All experiments were performed in duplicate. RNU6 and miR-484 served as internal reference genes for islet miRNA samples, and RNU6 alone served as the normaliser for EV miRNA samples. Relative fold expression against untreated control samples was calculated using the $2^{-\Delta\Delta}$Ct method (2).

Preparation of human plasma-derived EVs and RNA isolation

To isolate EVs, 500 µL of plasma sample was centrifuged at 1500 x g for 5 minutes to remove the residual cells and cellular debris. The supernatant was transferred to a fresh 1.5 mL tube, and 5 µL of de-fibrination reagent (Cat #TMEXO-1, System Biosciences, Palo Alto, CA) was added to the plasma to remove clotting factors prior to the isolation of EVs. The solution mixture was incubated at room temperature for 5 minutes with gentle mixing by flicking the tube. The sample was centrifuged at 9,391 x *g* for 5 minutes, and the supernatant was transferred to a clean tube. ExoQuick (Cat #EXOQ5A-1, System Biosciences, Palo Alto, CA) was added to the supernatant at a 1:4 ratio for 30 min at 4°C, followed by centrifugation at 1500 x g for 30 min. The supernatant was discarded without disturbing the pellet, and the isolated EVs were used for downstream applications.

Feature selection and machine learning analysis to identify predictive miRNA signatures

For feature selection, we used Learning Vector Quantification (LVQ) implemented in the 'caret' R package to compute the importance values of features, and any feature above a 0.7 importance value was considered significant and selected for machine learning analysis. Following variable selection, several supervised machine learning classifiers (including Decision Tree, Random Forest, and Support Vector Machine (SVM)) were used to predict cytokine-treated islet-derived EVs from untreated islet-derived EVs. The R packages 'rpart', 'randomforest', and 'e1701' were used for the decision tree, random forest, and SVM analysis, respectively. In the random forest analysis, the number of trees was set to 500.

Functional prediction of validated miRNAs

Functional enrichment analysis of the five selected miRNAs was carried out using DIANA miRPath v.3 (3). Specifically, TarBase was selected for target prediction, followed by gene ontology analysis. Biological process terms with FDR <0.05 were considered to be significant. Common and unique functional terms between all five miRNAs were identified.

LSPR-based biosensor construction

6-mercapto-1-hexanol was obtained from TCI Chemicals. RNase-free sterile water was purchased from Baxter Healthcare Corporation. RBS 35 Detergent, 18x18 mm glass coverslips, trifluoroacetic acid (TFA, 99%), and dichloromethane (DCM) were purchased from Fisher Scientific. Chloro(triethylphosphine) gold (I) (Et_3PAuCl, 97%) was purchased from Gelest Inc. Poly(methylhydrosiloxane) (PMHS, M_n = 1700-3300), triethylamine (TEA, 98%), hexamethylene tetramine (HMTA, ≥99.0%), 1,3,3-trimethyl-2-methylene indoline (TMMI, 97%), dimethyl

aminopyridine (DMAP, ≥98.0%), 1-hexanethiol (98%), and ACS grade acetonitrile (CH_3CN, 99.9%) were purchased from Sigma-Aldrich. 4-hydroxy benzoic acid (BA, 99%) and (N,N-dicyclohexylcarbdiimide (99%) were purchased from Acros Organics, and 3-Mercaptopropyl-trimethoxysilane (MPTMS, 94%) was purchased from Alfa Aesar. Ethanol (200 proof) (EtOH) was purchased from Decon Labs. Ethyl acetate (EtOAc) and Methanol (MeOH) were purchased from Pharmco. No-bottom 96-multiwell plates were purchased from Greiner Bio-One. Krazy glue was obtained from Office Depot. All water was purified using a Thermo Scientific Barnstead Nanopure system. All single-stranded oligonucleotides (ESM Table 5) for 155-5p (-ssDNA-155-5p), 802 (-ssDNA-802), 30c-1-3p (-ssDNA-30c-1-3p),124-3p (-ssDNA-124-3p), 146a-5p (-ssDNA-146a) and mature microRNAs (ESM Table 2), microRNA-155-5p, microRNA-802, microRNA-124-3p, microRNA-30c-1-3p and microRNA-146a-5p, were synthesized by Integrated DNA Technologies (IDT). The single-stranded oligonucleotide, mature microRNAs, and patient samples were stored at -80 °C. PBS buffer (pH = 7.2) was prepared using RNase-free sterile water. Ethanol and acetonitrile were purged with N_2 for 30 min prior to use.

<u>Silanisation of Glass Coverslips</u>: Glass coverslips with 18x18 mm dimensions were functionalised with MPTMS according to our previously published procedure (4–9). Briefly, glass coverslips were incubated in 10% (v/v) aqueous RBS detergent solution at 90°C and were sonicated for 15 min. Coverslips were then rinsed several times with nanopure water and incubated in a 1:1 (v/v) hydrochloric acid:methanol solution for 30 min at room temperature. Coverslips were then thoroughly rinsed with nanopure water, followed by drying in a vacuum oven at 60 °C for at least 12 hrs. Next, coverslips were brought to room temperature, followed by incubation in a 15% (v/v) solution of MPTMS in N_2 purged ethanol for 30 min. The coverslips were then sonicated at least three times in ethanol for 15 min each for washing. After completion of the ethanol washes, the

coverslips are dried in a vacuum oven at 120°C for at least 3 hrs. Finally, the MPTMS-functionalised coverslips were stored at 4°C for up to one week.

Synthesis of Gold Triangular Nanoprisms (Au TNPs): Au TNPs were chemically synthesized according to our previously published procedure with minor modifications (4–9). Briefly, gold salt, $Et_3PAu(I)Cl$ (18.4 mg, 0.05 mmol), was dissolved in 40 mL CH_3CN and stirred for 10 min at room temperature. Next, 38 µL (0.273 mmol) of TEA was added to the gold salt solution and heated to 38°C. At this point, 0.6 mL of PMHS was added, and the reaction was allowed to proceed with gentle stirring. During the reaction, the color of the solution changed from colorless to pink to light purple or blue. Once the solution displayed a stable LSPR dipole peak (λ_{LSPR}) position at 800 nm in CH_3CN, the reaction was stopped by removing the container from the hot plate. The solution was immediately centrifuged at 7547 x *g* for 10 sec, and the dark purple color solution was transferred to previously prepared MPTMS-functionalised 18x18 coverslips and incubated for exactly 1 hour, followed by rinsed with a copious amount of CH_3CN, dried under N_2 flow, and then stored under N_2 at 4°C for further use. Au TNP-bound glass coverslips were glued to a no-bottom 96-multiwell plate. This was accomplished by applying a small amount of super glue to the edge of 4 wells on the bottom side of the 96 well plate before placing a coverslip over the wells and gently pressing for 10 sec. Plates were allowed to dry for at least 2 hours at room temperature prior to use.

Synthesis of Receptor Binding Motif, SP-HT: The synthesis of spiropyran hexanethiol (SP-HT) was conducted using our published procedure with modifications (10). The synthesis consisted of three major steps: (1) synthesis of 3-formyl-4-hydroxybenzoic acid (BA-COOH), (2) synthesis of spiropyran carboxylic acid (SP-COOH), and (3) synthesis of spiropyran hexanethiol (SP-HT). (1)

Synthesis of BA-COOH: BA-COOH was synthesized utilising a Duff reaction, where 5 g (36.2 mmol) of 4-hydroxy-benzoic acid (BA) was added to a 100 mL 2-neck round bottom flask and mixed with 15 mL of trifluoroacetic acid (TFA) under nitrogen with stirring for 30 min at room temperature. Separately, 5 g (36.2 mmol) of hexamethylenetetramine (HMTA) was mixed with 15 mL of TFA. The mixture was then added dropwise to the round bottom flask containing the BA. After completion of the addition, the reaction vessel was transferred to a preheated 250°C oil bath and refluxed for 3 hours under N_2. ESI analysis was conducted to confirm BA was fully reacted by the disappearance of m/z 138. BA-COOH was then precipitated using 4 N hydrochloric acid (HCl) over 3 hours with vigorous stirring and recovered using vacuum filtration. The product was rinsed thoroughly with nanopure water and dried under vacuum overnight. ESI-MS analysis was used to confirm the presence of BA-COOH, MS (ESI): *m/z* = 166. (2) Synthesis of SP-COOH: 2.5 g (15.1 mmol) of synthesized BA-COOH was added to a 100 mL 2-neck round bottom flask under N_2, and 40 mL of purged ethanol was added and 3.8 mL (21.8 mmol) of 1,3,3-trimethyl-2-methylene indoline (TMMI) was obtained and added under N_2 with stirring. The reaction mixture was then placed in an oil bath and brought to reflux at 175°C for 3 hours. An ESI-MS analysis was conducted to determine that BA-COOH was no longer present by the disappearance of m/z 166. SP-COOH was purified using a silica gel column with a solvent gradient of dichloromethane (DCM) and methanol (MeOH). The SP-COOH product was obtained at 5% MeOH and the remainder at 10% MeOH. The fractions were concentrated using a rotatory evaporator (rotovap) and dried under a vacuum overnight. An ESI-MS product analysis was conducted to confirm the presence of SP-COOH, MS (ESI): *m/z* = 320. (3) Synthesis of spiropyran hexanethiol (SP-HT): This process utilised a DCC-NHS coupling of SP-COOH with 6-mercapto-1-hexanol to produce the final product, SP-HT. First, 5 g (15.7 mmol) of SP-COOH was added to 100 mL of purged dichloromethane (DCM) in a 250 mL 2-neck round bottom flask under nitrogen, and 2.5 g (18.8

mmol) of 6-mercapto-1-hexanol and 0.21 g (1.7 mmol) of dimethyl amino pyridine (DMAP) were both added directly to the reaction mixture. The reaction was stirred under N_2 in an ice bath until the internal temperature reached 0°C. Separately, 3.9 g (18.8 mmol) of N,N-dicyclohexylcarbdiimide (DCC) was dissolved in 100 mL of purged DCM. The DCC solution was added to the reaction flask dropwise over 40 min. The reaction then slowly reached room temperature with stirring and was allowed to react overnight under N_2. Before the reaction was stopped, an ESI analysis was conducted to determine that SP-COOH was no longer present by the disappearance of m/z 320. The reaction solution was then concentrated under rotovap and purified with a silica gel column. The column utilised a hexane (HEX) and ethyl acetate (EtOAc) gradient to obtain the desired SP-HT product. SP-HT was obtained at 40-50% EtOAc, and a 100% MeOH wash of the column was used to remove any remaining product. The fractions were concentrated using a rotovap and then dried under a vacuum overnight. An ESI-MS analysis of the product was conducted to confirm the presence and purity of SP-HT by the appearance of molecular weight *m/z* = 437, MS (ESI).

Fabrication of LSPR-Based Biosensors: Following SP-HT synthesis, a mixture of 75% SP-HT (0.025M) and 25% hexanethiol (HT) (0.025M) in acetonitrile (SP-HT:HT) was prepared. This solution was then poured into previously constructed Au TNP-containing 96 well plate where each well was filled with ~200 µL of SP-HT:HT solution and then incubated overnight. The wells were then rinsed with copious amounts of CH_3CN and dried with N_2 flow. The formation of mixed self-assembled monolayers (SAMs) of SP-HT:HT was confirmed by measuring the λ_{LSPR}. Utilising the photochromic nature of SP, the 96 well plates were then irradiated for ~5 min with a Kessil photoreaction light (KSPR160L-370) at 50% power. This process allowed the closed-ring spiropyran (SP) structure to be converted into the open-ring, zwitterionic merocyanine (MC) form.

λ_{LSPR} was used to confirm this photoisomerization. Once in the activated MC form, the now fully functional zwitterionic state could be utilised to bind the target receptor of choice, 300 µL of 10 µM -ssDNA-X (where X = -155-5p, -146a-5p, 802, -124-3p or -30c-1-3p), via a charge-charge interaction, as described below. The -ssDNA-X bound MC is considered a LSPR-based biosensor.

Spectroscopy and Microscopy Characterizations: Absorption and extinction spectra in the range of 700-1000 nm were collected with a SpectraMax M5 microplate reader from Molecular Devices, LLC. All absorption spectra were collected in PBS buffer (pH 7.2) to keep the bulk refractive index constant. A blank glass coverslip immersed in PBS buffer was used as a background, and an LSPR-based biosensor incubated in PBS buffer only was considered the blank reference. The chemically synthesized Au TNPs attached onto the MPTES-functionalised glass coverslips inside the 96 multiwell plate wells were characterized using a JEOL 7800F scanning electron microscopy (SEM) (ESM Fig. 5).

Development of Biomarker Calibration Plots: The LSPR extinction spectra of the LSPR-based biosensors were collected in PBS buffer to determine their dipole peak position (λ_{LSPR}) before and after analyte adsorption. Specifically constructed biosensors were incubated overnight in 300 µL of microRNA-X, where X = -155-5p, -146a-5p, -802, -124-3p, or -30c-1-3p solution of different concentrations (range of 100 nM to 100 aM) in 10% human plasma/PBS buffer solution (prepared via serial dilution) (ESM Table 6). Each analyte-bound biosensor was washed with PBS buffer to remove any non-specifically adsorbed biomolecules, the LSPR extinction spectra were collected, and the λ_{LSPR} was determined. The difference between the λ_{LSPR} before and after analyte adsorption ($\Delta\lambda_{LSPR}$) was used to develop calibration plots. The false positive analysis was conducted by incubating the LSPR-based biosensor with receptor molecule functionalization in a PBS buffer

solution without any analytes present. False negative analysis was conducted by incubating the biosensors without any receptor molecules present in the 10.0 nM biomarker solution (ESM Fig. 6).

Analysing Clinical Samples: Approximately 10 μL of human plasma-derived EV miRNA samples were diluted in 3 mL total of PBS buffer (pH 7.2), and then 300 μL was transferred to individual wells acting as a LSPR-based biosensor. Each biomarker was assayed in duplicate measurements. For islet cell-derived EV microRNA samples, we followed the same procedure. LSPR-based biosensors were incubated into miRNA samples overnight, followed by rinsing with excess nanopure water, and finally LSPR extinction spectra were collected. The λ_{LSPR} was determined for each well and the difference in the λ_{LSPR} from before (meaning after receptor -ssDNA-X attachment) and after patient sample microRNA adsorption to LSPR-based biosensor was determined ($\Delta\lambda_{LSPR}$) (ESM Table 7). The $\Delta\lambda_{LSPR}$ value was then plugged into the biomarker calibration plots to quantify the miRNA concentration for each patient sample as described below.

Data Processing and Statistical Analysis:

Processing UV-Vis Extinction Spectra: From the UV-Visible extinction spectra, λ_{LSPR} was determined through curve fitting using Origin software, and $\Delta\lambda_{LSPR}$ was derived by taking the difference between the λ_{LSPR} of the biosensors before and after attachment of the target miRNA analyte.

Processing Calibration Curves, Limit of Detection, and Concentration of Target miRNAs in Patient Plasma and Islet Cell Derived EV Lysate: As shown in ESM Fig. 3, calibration curves were developed by plotting $\Delta\lambda_{LSPR}$ vs. analyte concentration, with concentration being plotted in the logarithmic scale to investigate non-specific adsorption at a lower concentration range. The

calibration curve equation was determined through linear regression on Origin software. Finally, the LOD was determined using a "Z value" of the blank, where Z = mean + 3σ, when σ = standard deviation of the blank. This Z value is obtained from six $\Delta\lambda_{LSPR}$ measurements using six different biosensors. The Z value is then inserted as the "Y" coordinate of the calibration curve equation, allowing the LOD concentration to be determined (ESM Table 7). The concentrations of target miRNAs in patient samples were determined from the calibration curves developed in PBS buffer, with $\Delta\lambda_{LSPR}$ values and corresponding concentrations obtained from the average of six measurements. Each sample was independently analysed in duplicate, and each sample UV-Visible spectra was the average of three individual scans conducted by the plate reader.

EndoC-βH1 culture and EV analysis

EndoC-βH1 cells (Human Cell Design, France, RRID: CVCL_L909) were cultured on Matrigel-coated plates following previously described protocols (11,12). Cells were tested for the presence of mycoplasma infection for every 3-4 months using mycoplasma PCR detection kit (Cat# G238, ABM, Richmond, Canada). After 72 hours, the culture medium was replaced with fresh medium, and cells were treated with or without pro-inflammatory cytokines for 24 hours. At the end of the treatment period, conditioned media were collected and stored at −80 °C for subsequent extracellular vesicle (EV) isolation. EVs were isolated using differential ultra centrifugation (13). Total RNA was isolated from EVs using the miRNeasy RNA purification kit (Cat #217004, Qiagen), according to the manufacturer's instructions provided with the kit. RNA quality of each sample was measured using NanoDrop (Thermo Scientific, USA). cDNA was synthesized using the TaqMan Advance MicroRNA cDNA Synthesis Kit (Cat# A28007, Applied Biosystems, USA) and advance miRNA-specific TaqMan primers were used to determine the

expression of miRNAs using ddPCR, as described previously [44,45]. The data were expressed as copies/20 μL of PCR reaction.

BaseScope and RNAscope plus smRNA-RNA assays

Sample processing and pretreatment

To define tissue expression patterns of miR-155, single molecule fluorescent in-situ hybridisation (smFISH) was performed using a BaseScope duplex detection assay. Briefly, embedded pancreatic tissue sections from donors with T1D or AAb+ and non-diabetic control donors were received from the Network of Pancreatic Organ Donors (nPOD) tissue bank. The slides were baked at 60°C for 1 hour, and then the tissue sections were deparaffinized by incubating the slides twice with xylene for 5 minutes and 100% ethanol for 2 minutes at room temperature (RT). The slides were air dried at 60°C for 5 minutes, then moved to RT, and treated with hydrogen peroxide (ACD Cell Diagnostics, CA, USA) for 10 mins. After washing 5X with ddH2O, the slides were moved to a container with ddH2O at 99°C for 10 seconds, then immediately transferred to antigen-retrieval buffer (ACD Cell Diagnostics, CA, USA) at 99°C for 15 mins. Next, slides were washed with ddH2O for 15 secs at RT, followed by incubation with 100% ethanol for 3 mins and air-drying at RT. Sections were applied with a hydrophobic barrier (Immedge, Vector labs) and air-dried at RT. The slides were transferred to a humidifying chamber and treated with protease IV (ACD Cell Diagnostics, CA, USA) for 30 mins at 40°C in a HybEZ™ Oven (ACD Cell Diagnostics, CA, USA). Next, slides were washed 5X with ddH2O, and hybridisation was performed according to the manufacturer’s instructions using BaseScope duplex red assay probe sets.

BaseScope assay and immunofluorescence staining

The BaseScope duplex assay was performed per the instructions provided with the BaseScope kit protocol (ACD Cell Diagnostics, CA, USA). Because mature miRNAs are only ~22 nucleotides in length, we targeted to identify pre-miRNA expression in human tissue sections. Custom designed probe sets (ACD Cell Diagnostics, CA, USA) were generated to cover the regions of the human pre-miRNA sequence with a 1zz probe set covering 2-46 bp of pre-miR-155 (Cat# 18361A). Two control probe sets, PPIB-1zzas (Cat# 18282A, positive control) and bacterial DapB-1zz (Cat# 18267A, negative control), were used as internal controls to determine assay specificity. BaseScope Duplex Fast Red-B was used to detect pre-miR-155.

After the hybridisation process was completed, the slides were blocked with donkey serum in PBS for 30 minutes, followed by immunofluorescent staining for human insulin, as reported previously (12,14). Nuclei staining was performed using DAPI (ACD Cell Diagnostics, CA, USA), and the slides were mounted with a coverslip in Prolong Gold Antifade mounting medium (Invitrogen, USA).

RNAscope Plus smRNA-RNA Assay

To determine the efficiency of AAV8-RIP-miR-155 knockdown, tissues from AAV8-RIP-miRZip-155-5p or AAV8-RIP-miRZip-Scr-treated mice were harvested and assessed for mature miR-155 (ACD, Cat# 887751-S1 in combination with CD3e mRNA (ACD, Cat# 314721-C2). Hybridisation was performed using RNAscope plus smRNA-RNA assay kit (ACD, Cat#322785) according to the instructions provided by the manufactures protocol. Insulin (1:400 dilution, IR002, Dako, RRID: AB_2800361) protein staining was performed to identify beta cells and DAPI staining was used to identify nuclei.

Imaging

Imaging was performed using a Zeiss LSM800 confocal microscopy attached to an Airyscan detector (Carl Zeiss, Germany). All the images were acquired using a 60X oil objective with Z-stack, then the images were processed using an Airyscan processor to determine the individual foci of the pre-miRNAs. As described above, 4-5 islets were randomly selected from each slide for imaging and were used to quantify miRNA expression.

Machine learning-based smFISH image analysis.

Single-molecule quantification of miRNA in individual beta cells

Spatial quantification of pre-miRNA expression in single beta cells was composed of two steps: (i) smFISH image processing and (ii) machine learning-based classification. We followed the protocol described in our previous publication to process the smFISH images of pre-miRNA-155 in human pancreatic islets (14). In brief, we segmented single nuclei in the DAPI channel via a manual segmentation using ImageJ (15). Next, we identified the cell-cell boundaries and the cytoplasm of single cells by dilating the nuclear mask with 150 pixels (4.5 um). The identification of beta cells was accomplished by quantifying the fluorescent intensity in the corresponding insulin (Alexa 488) channel. Finally, the pre-miRNA was detected by a set of single particle detection algorithms, and the copy number was determined by normalising the smFISH foci intensity ($\mathrm{I_{foci}}$) by the intensity value of a single pre-miRNA ($\mathrm{I_{pre-miRNA}}$), ($\mathrm{N = I_{foci}/I_{pre-miRNA}}$). Each pre-miRNA localization was assigned to the nucleus or the cytoplasm of a given cell by encoding masked regions with a binary label and assigning the appropriate binary label based on transcript coordinates.

Extracting the feature of miRNA distributions in a single beta cell

A single-cell phenotyping algorithm with supervised machine learning was used to analyse the spatial organization of the pre-miRNA in single beta cells. Based on the preliminary processing and analysis of the pre-miRNA molecules, we utilised a series of descriptive methods, mathematical approaches, and statistical models to turn the spatial distribution of the pre-miRNA in a cell into an array of numbers. We then established a multi-dimensional feature library of the pre-miRNA in each beta cell. This feature library consists of two categories of pre-miRNA features in a single cell.

The first group of features are descriptive quantifications of individual pre-miRNAs in a single cell, which include the distance of each pre-miRNA to the nucleus centroid, the closest distance to the nucleic envelope, the distance of the pre-miRNA to its nearest neighbors, and other RNA localization-related properties (17). These values were averaged over all pre-miRNAs in a single cell, and each cell's mean value and standard deviation were kept as feature values. Meanwhile, we also included the spatial expression of the pre-miRNA as a feature, which includes the number of pre-miRNAs in the cytoplasm or nucleus.

The two other groups in the feature library were the collective quantification of pre-miRNA location in a single cell. Two categories of quantification were used. First, Ripley's K function was used to describe the clustering effect of the pre-miRNA in a single cell (18). Ripley's K function quantifies the degree of clustering at a specific radius r by selecting individual RNA particles, then counting the average number of surrounding RNA particles that lie within the circle with a radius r. We then normalised and shifted Ripley's K function into Ripley's H function, which transforms the expectation value of a completely random distribution to 0. Therefore, a positive result of Ripley's H function at a specific radius r indicates a clustered distribution at the corresponding radius, and a negative result shows a dispersed distribution. Features are extracted

by selecting Ripley's H function with specific intervals of radii. The extrema of Ripley's H function are also recorded as features, which have shown to be suitable indications for cluster sizes (19). Secondly, the number of pre-miRNAs located at the nucleus periphery was registered as boundary clustering features. This includes: i) nuclear pre-miRNA close to the nucleus envelope and ii) cytoplasmic pre-miRNA around the nucleus envelope. A similar pipeline has been developed to study sub-cellular RNA localization in simulated RNA images and cultured cells (20).

Training Network and Classification.

Following the extraction of features from cellular samples, the feature library was utilised for classification purposes, specifically distinguishing between healthy cells and cells obtained from organ donors with either AAb+ or established type 1 diabetes. This pairwise classification analysis was conducted to determine whether properties derived from RNA localization serve as potential indicators for type 1 diabetes or AAb+ status.

We used a random forest classifier as the feature-based classification method (21,22), which notably can determine the importance of each feature, thus allowing us to identify the RNA localization properties with the strongest relationship with the specific cell conditions. The imbalanced-learn Python library (23) was implemented to address the uneven number of cells in each sample by constructing each tree of the random forest classifier with a balanced bootstrap sample. The area under the receiver operating characteristic (ROC) curve (AUC) served as the metric for evaluating the classification performance of the classifiers. For the comparison of classification performance, two additional random forest classifiers were developed: one trained on miRNA expression features and the other on nucleus morphological features. The miRNA expression features comprised total miRNA count, nuclear miRNA count, and cytoplasmic

miRNA count. Nucleus morphological features were obtained using the ‘measure.regionprops()’ function from the scikit-image Python library, encompassing properties such as area, eccentricity, and convexity.

Immunohistochemistry

Pancreatic tissues from 12-week-old female NOD mice that were administered AAV8-RIP-miR-155 or AAV8-RIP-miR-Scr were harvested and fixed with 4% PFA overnight at 4°C and embedded in paraffin as described in our previous publications (14). Tissue sections were stained with rabbit anti-insulin antibody (1:400 dilution, Cell Signaling, 3014, RRID: AB_2126503) and counterstained with DAB peroxidase anti-rabbit IgG (Vector Lab). Images were acquired using a Zeiss slide scanner (Zeiss, Germany). For grading of insulitis, 5 μm pancreatic sections (25 μm apart) were stained and analysed for immune cell infiltration using Zeiss image analysis software.

Reference:

1. Love MI, Huber W, Anders S. Moderated estimation of fold change and dispersion for RNA-seq data with DESeq2. Genome Biol. 2014 Dec 5;15(12):550. doi:10.1186/s13059-014-0550-8

2. Livak KJ, Schmittgen TD. Analysis of relative gene expression data using real-time quantitative PCR and the 2(-Delta Delta C(T)) Method. Methods San Diego Calif. 2001 Dec;25(4):402–8. doi:10.1006/meth.2001.1262 PubMed PMID: 11846609.

3. Vlachos IS, Zagganas K, Paraskevopoulou MD, Georgakilas G, Karagkouni D, Vergoulis T, et al. DIANA-miRPath v3.0: deciphering microRNA function with experimental support. Nucleic Acids Res. 2015 Jul 1;43(Web Server issue):W460–6. doi:10.1093/nar/gkv403 PubMed PMID: 25977294; PubMed Central PMCID: PMC4489228.

4. Joshi GK, McClory PJ, Muhoberac BB, Kumbhar A, Smith KA, Sardar R. Designing Efficient Localized Surface Plasmon Resonance-Based Sensing Platforms: Optimization of Sensor Response by Controlling the Edge Length of Gold Nanoprisms. J Phys Chem C. 2012 Oct 4;116(39):20990–1000. doi:10.1021/jp302674h

5. Joshi GK, Smith KA, Johnson MA, Sardar R. Temperature-Controlled Reversible Localized Surface Plasmon Resonance Response of Polymer-Functionalized Gold Nanoprisms in the Solid State. J Phys Chem C. 2013 Dec 12;117(49):26228–37. doi:10.1021/jp409264w

6. Joshi GK, Deitz-McElyea S, Johnson M, Mali S, Korc M, Sardar R. Highly Specific Plasmonic Biosensors for Ultrasensitive MicroRNA Detection in Plasma from Pancreatic Cancer Patients. Nano Lett. 2014 Dec 10;14(12):6955–63. doi:10.1021/nl503220s

7. Joshi GK, Deitz-McElyea S, Liyanage T, Lawrence K, Mali S, Sardar R, et al. Label-Free Nanoplasmonic-Based Short Noncoding RNA Sensing at Attomolar Concentrations Allows for Quantitative and Highly Specific Assay of MicroRNA-10b in Biological Fluids and Circulating Exosomes. ACS Nano. 2015 Nov 24;9(11):11075–89. doi:10.1021/acsnano.5b04527

8. Liyanage T, Masterson AN, Oyem HH, Kaimakliotis H, Nguyen H, Sardar R. Plasmoelectronic-Based Ultrasensitive Assay of Tumor Suppressor microRNAs Directly in Patient Plasma: Design of Highly Specific Early Cancer Diagnostic Technology. Anal Chem. 2019 Feb 5;91(3):1894–903. doi:10.1021/acs.analchem.8b03768

9. Masterson AN, Liyanage T, Kaimakliotis H, Gholami Derami H, Deiss F, Sardar R. Bottom-Up Fabrication of Plasmonic Nanoantenna-Based High-throughput Multiplexing Biosensors for Ultrasensitive Detection of microRNAs Directly from Cancer Patients' Plasma. Anal Chem. 2020 Jul 7;92(13):9295–304. doi:10.1021/acs.analchem.0c01639

10. Hati S, Langlais SR, Masterson AN, Liyanage T, Muhoberac BB, Kaimakliotis H, et al. Photoswitchable Machine-Engineered Plasmonic Nanosystem with High Optical Response for Ultrasensitive Detection of microRNAs and Proteins Adaptively. Anal Chem. 2021 Oct 19;93(41):13935–44. doi:10.1021/acs.analchem.1c02990

11. Ravassard P, Hazhouz Y, Pechberty S, Bricout-Neveu E, Armanet M, Czernichow P, et al. A genetically engineered human pancreatic β cell line exhibiting glucose-inducible insulin secretion. J Clin Invest. 2011 Sep 1;121(9):3589–97. doi:10.1172/JCI58447 PubMed PMID: 21865645; PubMed Central PMCID: PMC3163974.

12. Nakayasu ES, Syed F, Tersey SA, Gritsenko MA, Mitchell HD, Chan CY, et al. Comprehensive Proteomics Analysis of Stressed Human Islets Identifies GDF15 as a Target for Type 1 Diabetes Intervention. Cell Metab. 2020 Jan;S1550413119306709. doi:10.1016/j.cmet.2019.12.005

13. Cianciaruso C, Phelps EA, Pasquier M, Hamelin R, Demurtas D, Ahmed MA, et al. Primary Human and Rat β-Cells Release the Intracellular Autoantigens GAD65, IA-2, and Proinsulin in Exosomes Together With Cytokine-Induced Enhancers of Immunity. Diabetes. 2017 Feb 1;66(2):2. doi:10.2337/db16-0671

14. Wu W, Syed F, Simpson E, Lee CC, Liu J, Chang G, et al. The Impact of Pro-Inflammatory Cytokines on Alternative Splicing Patterns in Human Islets. Diabetes. 2021 Oct 21. doi:10.2337/db20-0847 PubMed PMID: 34697029.

15. Soille P, Vincent LM. Determining watersheds in digital pictures via flooding simulations. In: Visual Communications and Image Processing ’90: Fifth in a Series [Internet]. SPIE; 1990 [cited 2022 Apr 29]. p. 240–50. Available from: https://www.spiedigitallibrary.org/conference-proceedings-of-spie/1360/0000/Determining-watersheds-in-digital-pictures-via-flooding-simulations/10.1117/12.24211.full doi:10.1117/12.24211

16. McQuin C, Goodman A, Chernyshev V, Kamentsky L, Cimini BA, Karhohs KW, et al. CellProfiler 3.0: Next-generation image processing for biology. PLoS Biol. 2018 Jul;16(7):e2005970. doi:10.1371/journal.pbio.2005970 PubMed PMID: 29969450; PubMed Central PMCID: PMC6029841.

17. Battich N, Stoeger T, Pelkmans L. Image-based transcriptomics in thousands of single human cells at single-molecule resolution. Nat Methods. 2013 Nov;10(11):11. doi:10.1038/nmeth.2657

18. Ripley BD. Modelling Spatial Patterns. J R Stat Soc Ser B Methodol. 1977;39(2):172–92. doi:10.1111/j.2517-6161.1977.tb01615.x

19. Kiskowski MA, Hancock JF, Kenworthy AK. On the use of Ripley’s K-function and its derivatives to analyze domain size. Biophys J. 2009 Aug 19;97(4):1095–103. doi:10.1016/j.bpj.2009.05.039 PubMed PMID: 19686657; PubMed Central PMCID: PMC2726315.

20. Samacoits A, Chouaib R, Safieddine A, Traboulsi AM, Ouyang W, Zimmer C, et al. A computational framework to study sub-cellular RNA localization. Nat Commun. 2018 Nov 2;9(1):4584. doi:10.1038/s41467-018-06868-w PubMed PMID: 30389932; PubMed Central PMCID: PMC6214940.

21. Breiman L. Random Forests. Mach Learn. 2001 Oct 1;45(1):5–32. doi:10.1023/A:1010933404324

22. Ho TK. The random subspace method for constructing decision forests. IEEE Trans Pattern Anal Mach Intell. 1998 Aug;20(8):832–44. doi:10.1109/34.709601

23. Lemaitre G, Nogueira F, Aridas CK. Imbalanced-learn: A Python Toolbox to Tackle the Curse of Imbalanced Datasets in Machine Learning [Internet]. arXiv; 2016 [cited 2022 Dec 8]. Available from: http://arxiv.org/abs/1609.06570 doi:10.48550/arXiv.1609.06570

ESM TABLES

ESM Table 1. Human islet checklist

ESM Table 1 has been uploaded as a separate file, as it is an Excel file.

ESM Table 2. microRNA sequences that were used to generate calibration curves

Name	Nucleic Acid microRNA Sequence
miR-155-5p	5’- UUA AUG CUA AUC GUG AUA GGG GUU -3’
miR-146a-5p	5’ – UGA GAA CUG AAU UCC AUG GGU U – 3’
miR-802	5’ - CAG UAA CAA AGA UUC AUC CUU GU – 3’
miR-124-3p	5’ - UAA GGC ACG CGG UGA AUG CCA A – 3’
miR-30c-1-3p	5’ – CUG GGA GAG GGU UGU UUA CUC C – 3’

ESM Table 3. Descriptive statistics

ESM Table 3A. Descriptive statistics of islets

Sample name	Batch	Treatment	Total reads	Total aligned reads	Total unaligned reads	Mapped to miRNA
Sample 1_Cytokine	1	Cytokine	2,054,411	1,300,648	753,764	660,907
Sample 2_Cytokine	1	Cytokine	3,124,061	2,127,798	996,377	1,091,088
Sample 3_Cytokine	1	Cytokine	2,591,878	1,694,829	897,065	656,258
Sample 4_Cytokine	1	Cytokine	2,326,519	1,664,624	661,908	622,900
Sample 5_Cytokine	2	Cytokine	14,664,373	12,065,846	2,599,253	2,451,914
Sample 6_Cytokine	2	Cytokine	13,377,553	9,496,725	3,880,288	2,272,287
Sample 7_Cytokine	2	Cytokine	8,823,179	5,180,088	3,642,957	1,571,907
Sample 8_Cytokine	2	Cytokine	14,697,258	10,689,316	4,007,947	2,038,327
Sample 9_Cytokine	2	Cytokine	15,187,948	10,303,504	4,883,966	1,853,587
Sample 10_Cytokine	2	Cytokine	11,132,752	6,025,045	5,107,693	1,754,292
Sample 1_untreated	1	Untreated	1,854,137	945,981	908,195	498,742
Sample 2_untreated	1	Untreated	2,352,048	1,179,317	1,172,746	582,585
Sample 3_untreated	1	Untreated	2,401,487	1,357,801	1,043,636	553,268
Sample 4_untreated	1	Untreated	2,349,003	1,757,994	591,095	928,985
Sample 5_untreated	2	Untreated	13,132,128	9,988,297	3,144,136	2,616,396
Sample 6_untreated	2	Untreated	14,963,188	11,017,395	3,946,395	1,871,802
Sample 7_untreated	2	Untreated	16,269,364	11,944,967	4,325,159	1,889,477
Sample 8_untreated	2	Untreated	12,869,993	8,602,303	4,267,563	2,316,388
Sample 9_untreated	2	Untreated	6,662,954	4,308,266	2,354,925	839,713
Sample 10_untreated	2	Untreated	10,287,444	6,178,639	4,108,689	1,332,384

ESM Table 3B. Descriptive statistics of EVs

Sample name	Batch	Treatment	Total reads	Total aligned	Total unaligned	Mapped to miRNAs
Sample 1_Cytokine	2	Cytokine	4,264,849	2,863,420	1,401,544	1,176,172
Sample 2_Cytokine	2	Cytokine	2,135,118	1,082,718	1,052,459	255,727
Sample 3_Cytokine	2	Cytokine	4,055,979	2,141,963	1,913,961	557,978
Sample 4_Cytokine	2	Cytokine	3,356,133	2,013,344	1,342,806	569,917
Sample 5_Cytokine	2	Cytokine	3,697,693	1,974,198	1,723,462	510,295
Sample 6_Cytokine	1	Cytokine	3,373,475	1,981,579	1,391,753	499,652
Sample 7_Cytokine	2	Cytokine	4,372,133	1,929,860	2,442,437	372,539
Sample 8_Cytokine	1	Cytokine	2,199,842	1,413,398	786,369	393,698
Sample 9_Cytokine	2	Cytokine	5,132,436	3,181,084	1,951,577	1,045,087
Sample 10_Cytokine	1	Cytokine	1,031,149	709,327	321,805	167,226
Sample 11_Cytokine	2	Cytokine	3,757,155	2,037,129	1,719,916	786,489
Sample 12_Cytokine	1	Cytokine	3,919,830	2,854,420	1,065,575	806,124
Sample 13_Cytokine	2	Cytokine	4,000,971	2,270,551	1,730,429	1,084,639
Sample 14_Cytokine	2	Cytokine	3,606,848	1,511,991	2,094,851	190,957
Sample 1_untreated	2	Healthy	4,095,296	3,139,454	955,906	1,493,497
Sample 2_untreated	2	Healthy	2,222,263	1,080,686	1,141,621	227,017
Sample 3_untreated	2	Healthy	3,136,268	1,767,914	1,368,472	414,766
Sample 4_untreated	2	Healthy	2,200,147	1,184,119	1,016,069	425,164
Sample 5_untreated	2	Healthy	2,687,655	1,265,886	1,421,779	275,131
Sample 6_untreated	1	Healthy	2,482,904	1,052,503	1,430,455	211,606
Sample 7_untreated	2	Healthy	2,726,129	1,270,376	1,455,826	228,409
Sample 8_untreated	1	Healthy	2,013,477	1,265,470	747,934	406,393
Sample 9_untreated	2	Healthy	3,139,816	1,711,828	1,428,073	669,261
Sample 10_untreated	1	Healthy	1,028,515	628,423	400,084	98,767
Sample 11_untreated	2	Healthy	3,744,869	1,717,022	2,027,995	424,485
Sample 12_untreated	1	Healthy	4,428,561	2,899,822	1,528,622	981,547
Sample 13_untreated	2	Healthy	3,525,663	1,623,568	1,902,017	491,683
Sample 14_untreated	2	Healthy	3,769,590	1,778,493	1,990,927	240,566

ESM Table 4. Differentially expressed miRNAs in islets and EVs. miRNAs with a fold change of ≥ 1.5 and $p < 0.05$ were considered as differentially expressed. 20 miRNAs and 14 miRNAs were identified as differentially expressed from islets (left panel) and extracellular vesicles (right panel), respectively. miRNAs highlighted in red indicate miRNAs common between islets and EVs.

Differentially expressed miRNAs in islets			Differentially expressed miRNAs in EVs		
**miRNA**	**Linear scale fold change**	**p-value**	**miRNA**	**Linear scale fold change**	**p-value**
hsa-miR-155-5p	7.13	4.07E-40	hsa-miR-155-5p	2.59	0.0001
hsa-miR-6891-5p	4.58	2.41E-08	hsa-miR-1226-3p	2.02	0.0178
hsa-miR-4640-5p	2.47	0.0002	hsa-miR-802	1.63	1.64E-05
hsa-miR-146a-5p	2.4	1.15E-09	hsa-miR-146a-5p	1.63	2.97E-05
hsa-miR-205-5p	2.29	1.13E-07	hsa-miR-31-3p	1.6	0.0085
hsa-miR-338-3p	2.15	0.0113	hsa-miR-30c-1-3p	1.6	0.0480
hsa-miR-21-3p	2.08	3.59E-08	hsa-miR-193b-5p	1.56	0.0031
hsa-miR-27a-5p	2.03	0.0052	hsa-miR-193b-3p	1.53	0.0004
hsa-miR-6743-3p	1.68	0.0479	hsa-miR-148a-3p	1.52	4.30E-05
hsa-miR-23a-5p	1.63	0.0008	hsa-miR-217	1.51	0.0001
hsa-miR-431-5p	1.61	0.0006	hsa-miR-216a-3p	1.51	0.0085
hsa-miR-363-3p	1.57	0.0260	hsa-miR-4485	-1.67	0.0111
hsa-miR-194-3p	1.52	0.0047	hsa-miR-210-5p	-1.8	0.0436
hsa-miR-147b	1.52	0.0080	hsa-miR-124-3p	-2.22	0.0092
hsa-miR-543	1.5	0.0001			
hsa-miR-33a-5p	-1.55	0.0113			
hsa-miR-181a-2-3p	-1.8	3.14E-05			
hsa-miR-335-3p	-1.88	0.0094			
hsa-miR-675-5p	-2.65	5.36E-06			
hsa-miR-338-5p	-3.12	0.0042			

ESM Table 5. Single stranded DNA (*-ssDNA)* oligomer sequences used in this study

Name	Nucleic Acid -ssDNA Sequence
-ssDNA-155-5p	5’ - AAC CCC TAT CAC GAT TAG CAT TAA – 3’
-ssDNA-146a-5p	5’ – AAC CCA TGG AAT TCA GTT CTC A – 3’
-ssDNA-802	5’ - ACA AGG ATG AAT CTT TGT TAC TG – 3’
-ssDNA-124-3p	5’ - TTG GCA TTC ACC GCG TGC CTT A – 3’
-ssDNA-30c-1-3p	5’ - GGA GTA AAC AAC CCT CTC CCA G – 3’

ESM Table 6. ***Δλ***

microRNA	microRNA Concentration (nM)	Average $\Delta\lambda_{LSPR}$ (nm)	Standard Deviation (nm)
miR-155-5p	100	11.8	0.4
	10	10.0	0.7
	1	8.8	0.4
	0.1	7.3	0.4
	0.01	6.0	0.1
	0.001	5.3	0.4
	0.0001	4.0	0.1
	0.00001	3.0	0.1
	0.000001	2.5	0.1
	0.0000001	1.5	0.1
miR-124-3p	100	10.5	0.7
	10	9.3	0.4
	1	8.0	0.1
	0.1	7.4	0.2
	0.01	6.0	0.7
	0.001	5.3	0.4
	0.0001	4.0	0.4
	0.00001	3.1	0.5
	0.000001	2.5	0.1
	0.0000001	1.6	0.1
miR-802	100	9.8	0.1
	10	8.4	0.2
	1	7.3	0.4
	0.1	6.3	0.7
	0.01	5.5	0.1
	0.001	4.5	0.4
	0.0001	3.4	0.5
	0.00001	2.5	0.4
	0.000001	1.8	0.1
	0.0000001	1.0	0.4
miR-146a-5p	100	10.1	0.2
	10	9.0	0.1
	1	8.1	0.5
	0.1	7.0	0.1
	0.01	6.1	0.5
	0.001	4.9	0.2
	0.0001	4.0	0.1
	0.00001	3.1	0.2
	0.000001	2.3	0.4
	0.0000001	1.5	0.1
miR-30c-1-3p	100	11.1	0.2
	10	10.0	0.4
	1	9.1	0.2
	0.1	7.9	0.2
	0.01	6.8	0.1
	0.001	5.5	0.1
	0.0001	4.5	0.4
	0.00001	3.1	0.2
	0.000001	2.3	0.4
	0.0000001	1.5	0.4

ESM Table 7. $\Delta\lambda_{LSPR}$ responses of spiropyran based plasmonic nanosensors for nucleic acid -ssDNA attachment and microRNA targets at 100 nM concentrations

Nucleic Acid Gene	Average $\Delta\lambda_{LSPR}$ (nm) after attachment of corresponding 10 µM -ssDNA receptor	Average $\Delta\lambda_{LSPR}$ (nm) after addition of 100 nM Nucleic Acid microRNA Target
**hsa-155-5p gene**	*+12.0 (±0.9)*	*+11.8 (±0.4)*
**hsa-146a-5p gene**	*+11.6 (±1.9)*	*+10.1 (±0.2)*
**hsa-802 Gene**	*+10.9 (±1.7)*	*+9.8 (±0.1)*
**hsa-124-3p Gene**	*+11.8 (±1.0)*	*+10.5 (±0.7)*
**hsa-30c-1-3p Gene**	*+12.2 (±1.6)*	*+11.1 (±0.2)*

ESM FIGURES and FIGURE LEGENDS

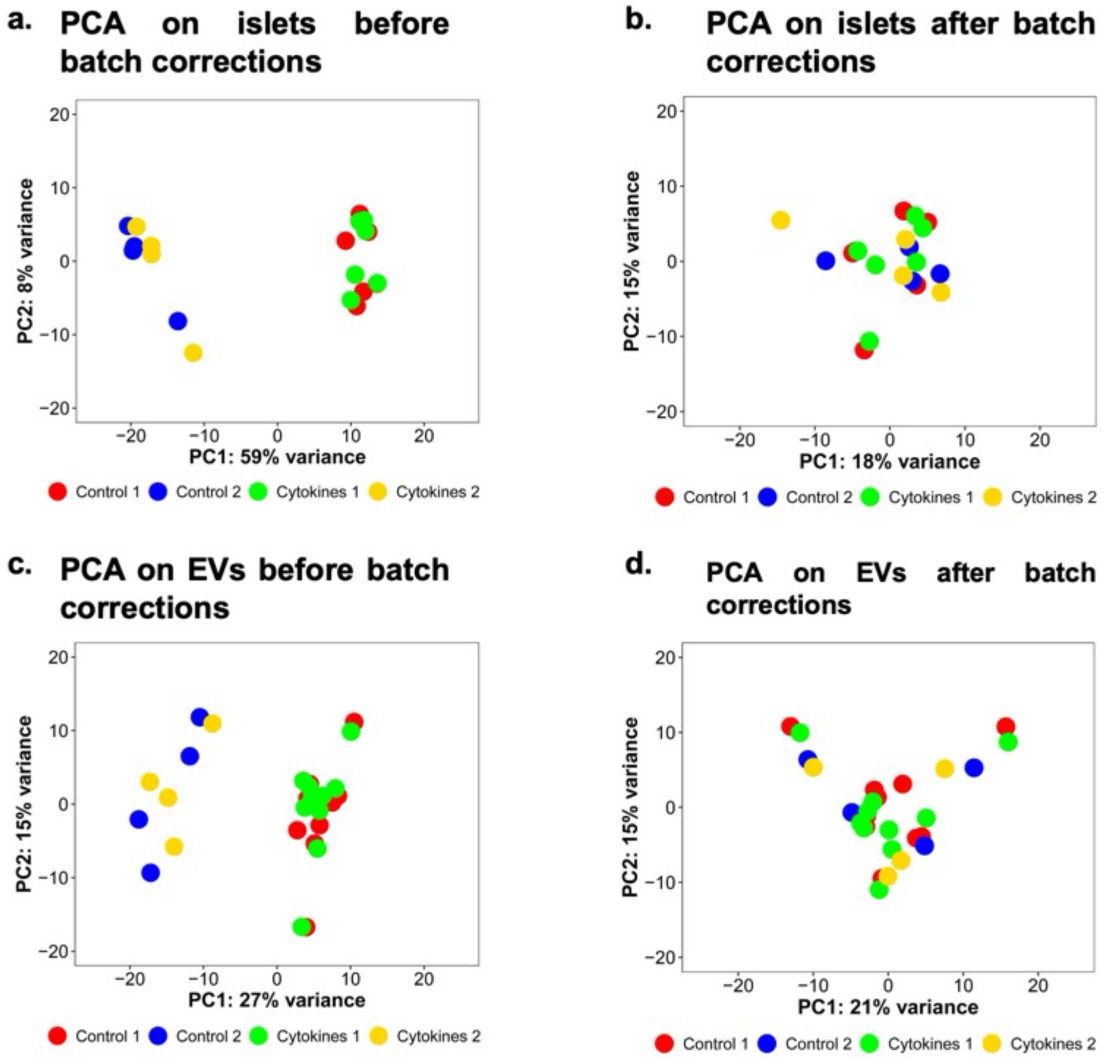

ESM Figure 1. Principal component analysis of small RNA sequencing performed on islets and extracellular vesicles. In both (a) islets and (c) EVs, principal component analysis (PCA) using variance stabilized counts showed the presence of batch effects, as the samples clustered based on batches. PCA clustering using batch corrected variance stabilized counts revealed no clustering of samples based on batches, confirming removal of batch effects from both (b) islets and (d) EVs.

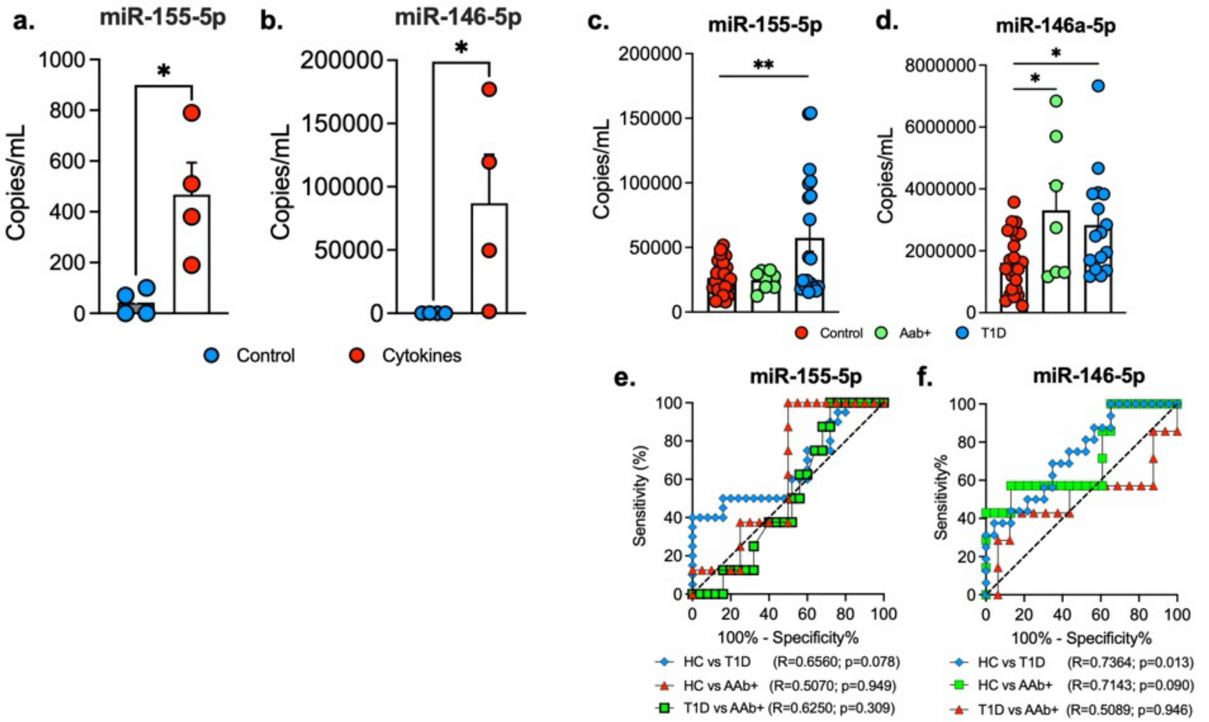

ESM Figure 2. Droplet digital PCR (ddPCR) analysis of miRNAs in EVs isolated from EndoC-βH1 cells and human plasma samples. Expression of **(a)** miR-155-5p and **(b)** miR-146-5p miRNAs measured in EVs isolated from EndoC-βH1 cells treated with or without proinflammatory cytokines. Bar graphs (**c-d**) and their associated ROC curves **(e-f)** for miR-155-5p and miR-146-5p measured using ddPCR in plasma collected from the same donors used in the LSPR assays. *p<0.05, **p<0.01.

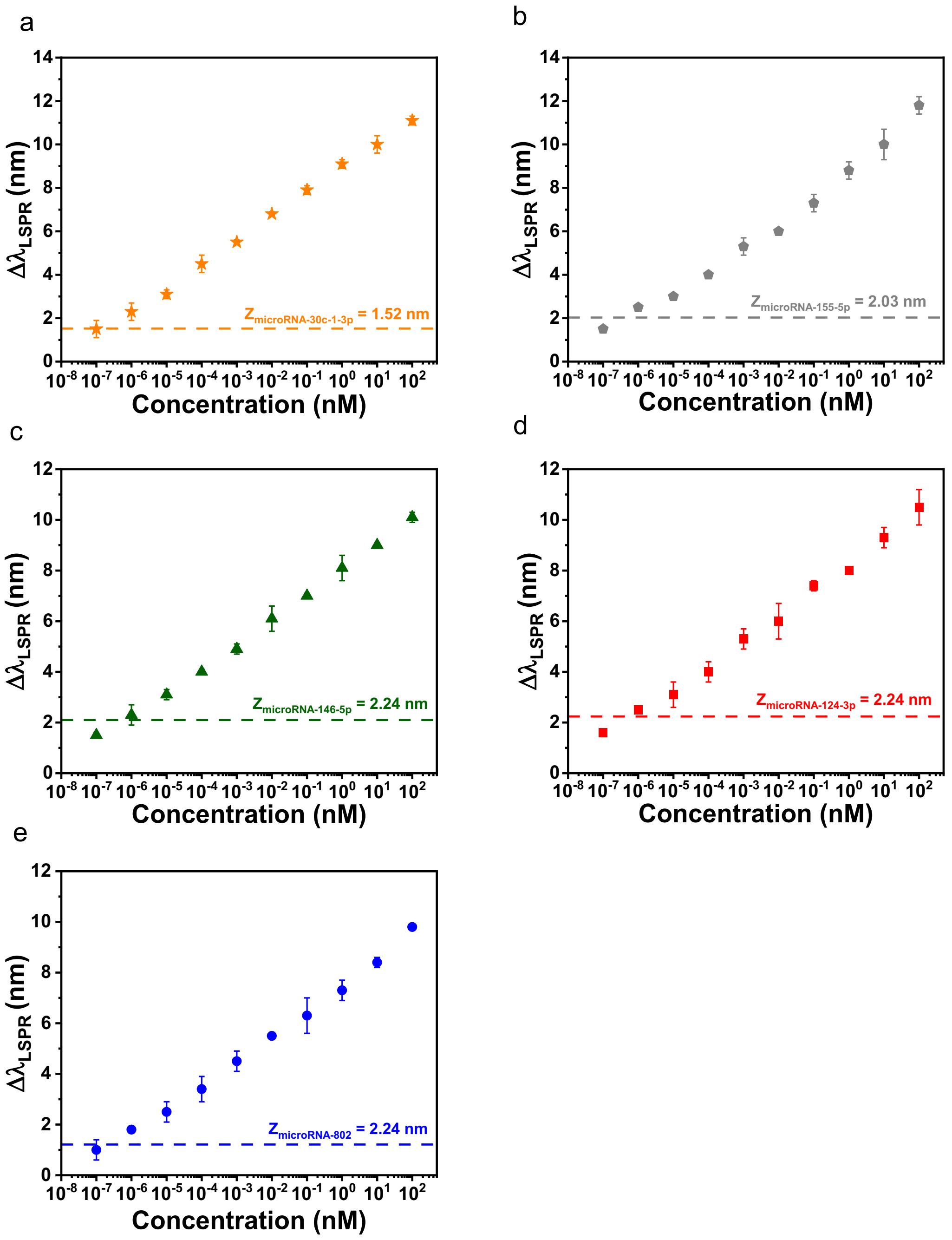

ESM Figure 3. Calibration curve generated using synthetic microRNAs. Calibration curve of microRNAs (miR) representing $\Delta\lambda_{LSPR}$ versus concentration (nM) being plotted in the logarithm scale as described above; (a) miR-30c-1-3p (orange star); (b) mir-155-5p (gray pentagon); (c) miR-146a-5p (green triangle); (d) miR-124-3p (red square); and (e) miR-802 (blue circle).

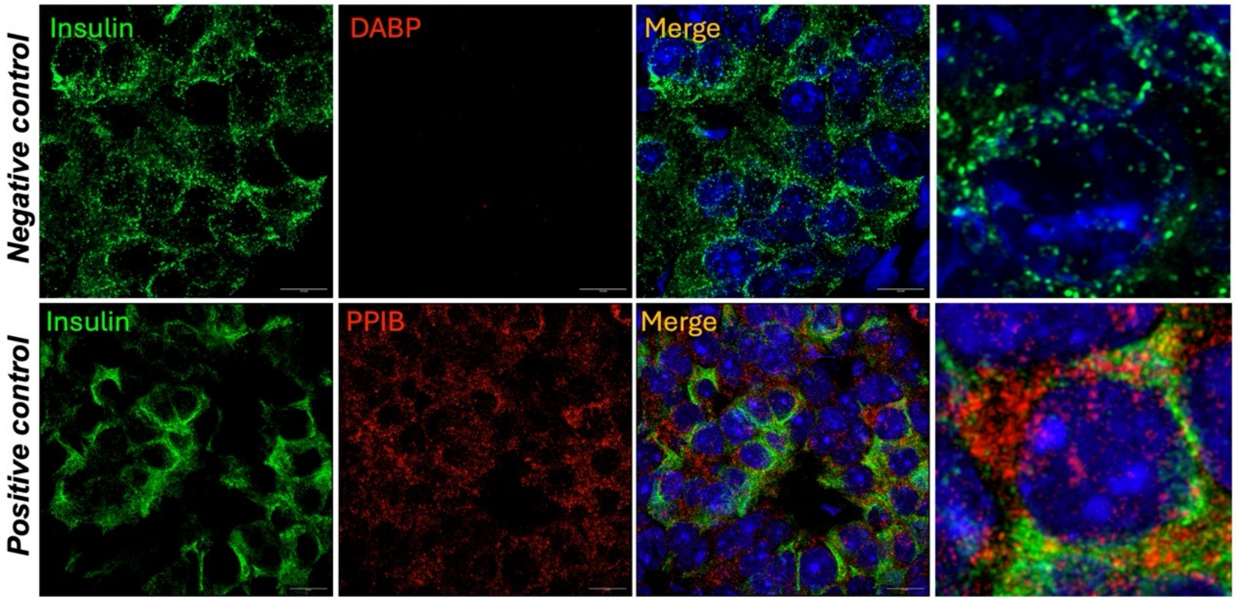

ESM Figure 4. Representative images showing positive and negative controls for smFISH analysis. Representative images of human pancreatic tissue sections that are hybridised and stained for positive and (PPIB) and negative controls (DABP). Scale bar: 10 µm.

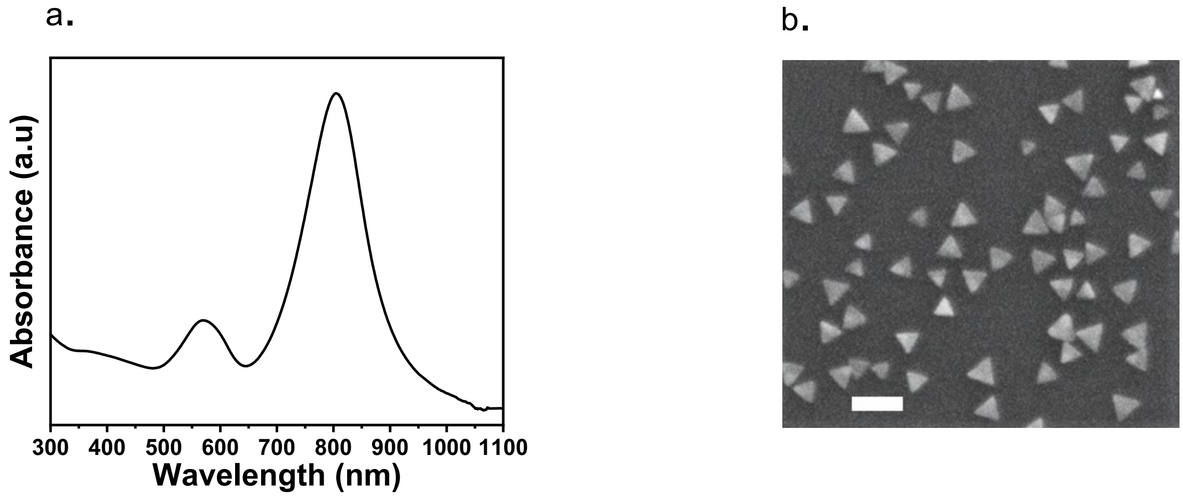

ESM Figure 5. UV-visible absorption spectra and scanning electron microscope image of Au TNPs. (a) UV-visible absorption spectra of Au TNP solution in ACN (black, 801.0 nm); (b) A corresponding representative scanning electron microscope image of Au TNPs with a length of 42 nm and thickness of 8 nm. The scale bar is 100 nm. The dimension of the Au TNP was determined using ImageJ software.

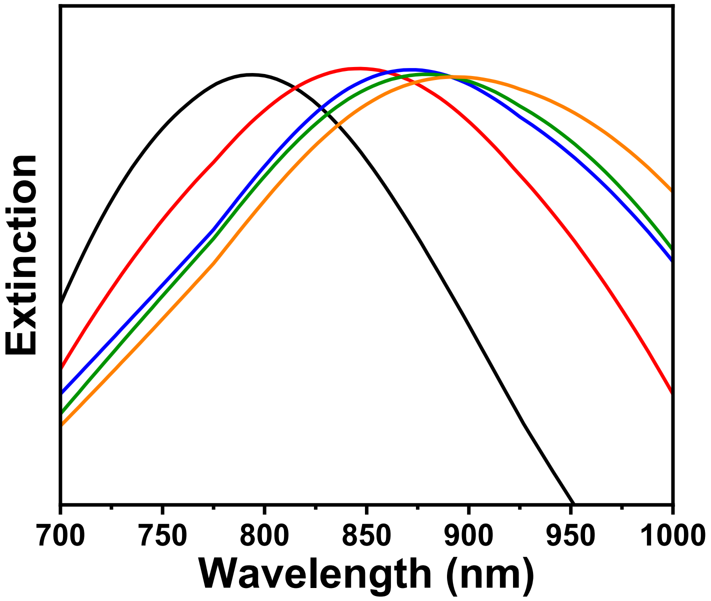

ESM Figure 6. Representative UV-vis extinction spectra showing Au TNP, SP-HT:HT, MC-HT:HT, -ssDNA-155-5p attachment and microRNA-155-5p hybridisation. Representative UV-vis extinction spectra showing Au TNPs before (black, λ_{LSPR} = 799.9 nm), after functionalisation with 75%:25% SP-HT:HT SAMs (red, λ_{LSPR} = 853.0 nm), after exposure to UV light (blue, λ_{LSPR} = 874.0 nm), followed by incubation in 10 μM -ssDNA-155-5p receptor in PBS buffer solution (green, λ_{LSPR} = 886.2 nm) and, lastly, incubation in 100 nM microRNA-155-5p in 10% human plasma (orange, 898.0 nm).
